# Integrated surveillance, virus isolation and phylogenetic characterization of Crimean-Congo hemorrhagic fever virus in Central Kazakhstan

**DOI:** 10.3389/fvets.2026.1879322

**Published:** 2026-07-16

**Authors:** Zhumagali Koshemetov, Kayyrzhan Baizhanov, Nurgul Orazymbetova, Madina Kaukarbayeva, Akezhan Issakhan, Bakyt Umuraliyev, Orazbek Serikbayov, Nurlan Kozhabergenov, Madina Seisenbayeva, Kuandyk Zhugunissov, Kydyrbay Maikhin, Abdikalyk Abishov, Zhanat Kondibayeva, Aliya Adalbekova, Yerbol Bulatov, Dariya Toktyrova

**Affiliations:** 1 Research Institute for Biological Safety Problems, National Holding QazBioPharm, Gvardeyskiy, Kazakhstan; 2 JSC National Holding QazBioPharm, Astana, Kazakhstan; 3 Almaty Branch of the National Reference Center for Veterinary Medicine, Almaty, Kazakhstan; 4 LLP Research and Production Center DiaVak-ABN, Almaty, Kazakhstan

**Keywords:** Crimean-Congo hemorrhagic fever virus, ixodid ticks, phylogenetic analysis, surveillance, transboundary zoonotic infections

## Abstract

This study investigated the circulation of Crimean-Congo hemorrhagic fever virus (CCHFV) among livestock and ixodid ticks in the Karaganda and Ulytau regions of Central Kazakhstan and characterized the detected viral isolate. A targeted cross-sectional surveillance study was conducted during the spring–summer period of 2025 in the Shet, Aktogay, Ulytau, and Zhanaarka districts, as well as in the cities of Zhezkazgan, Satpayev, and Karazhal, selected because of pasture-based livestock management, livestock movement routes, and ecological suitability for ixodid tick populations. In total, 700 whole blood and 700 serum samples were collected from cattle and small ruminants. Additionally, 127 ticks were collected by flagging, direct removal from animals, and manual sampling and were identified morphologically. CCHFV RNA was detected using RT-qPCR, while anti-CCHFV IgG antibodies were detected by ELISA. Virus isolation was performed in BHK-21/13, Vero, CV-1, and MARC-145 cell lines. Viral RNA was not detected in livestock blood samples; however, one of eight tick pools collected in the Mibulak rural district tested positive by RT-qPCR. Anti-CCHFV IgG antibodies were detected in eight serum samples, including four originating from the same locality. A pronounced cytopathic effect was observed in BHK-21/13 and Vero cells on days 4–5 post-infection. Phylogenetic analysis of the S segment demonstrated that isolate CCHF/2025/Mibulak/KZ belongs to the Asia-2 genotype and clusters with strains previously reported from China, Kazakhstan, and Uzbekistan. Taken together, these findings support evidence of localized CCHFV circulation in Central Kazakhstan and highlight the importance of sustained integrated One Health surveillance for early detection and prevention of human infection.

## 1 Introduction

Crimean-Congo hemorrhagic fever virus (CCHFV) belongs to the family *Nairoviridae* within the order *Bunyavirales*. It is an enveloped negative-sense single-stranded RNA virus with a tripartite genome consisting of the S, M, and L segments (1, 2). The S segment encodes the nucleocapsid protein, the M segment encodes the viral glycoproteins Gn and Gc, and the L segment encodes the RNA-dependent RNA polymerase (3).

Crimean-Congo hemorrhagic fever (CCHF) is a tick-borne zoonotic disease with substantial veterinary and public health importance. In domestic and wild animals, infection is usually asymptomatic; however, infected animals play an important role as amplifying hosts within the epidemiological cycle of the virus (4, 5). Human infection occurs primarily through tick bites or contacts with blood and tissues of infected animals (1). Ticks of the genus *Hyalomma*, particularly *Hyalomma marginatum*, are considered the principal vectors and reservoirs of CCHFV (6, 7). Other tick genera, including *Rhipicephalus* and *Dermacentor*, may also contribute to virus maintenance and transmission (7–9). In humans, the potential for onward transmission is considered very limited; humans are regarded as dead-end hosts, as the virus is not transmitted from infected individuals back to ticks (1). In addition to ticks, various animal species may play a role in the epidemiological cycle. Antibodies to CCHFV have been detected in cattle, sheep, goats, camels, horses, and wild ungulates, indicating widespread subclinical exposure among animal hosts (10, 11).

In humans, CCHF is characterized by acute febrile illness that may progress to severe hemorrhagic manifestations with high case fatality rates in hospitalized patients (12). A substantial proportion of infections are believed to remain asymptomatic or undiagnosed, complicating epidemiological surveillance and accurate disease burden assessment (12, 13). CCHFV is endemic in large parts of Africa, the Middle East, Asia, and southeastern Europe, largely corresponding to the geographic distribution of *Hyalomma* ticks (13–15).

In Kazakhstan, CCHF is considered an endemic disease, with natural foci primarily located in the southern regions, including Zhambyl, Turkestan, and Kyzylorda. Since the first documented cases in 1948, human infections have been reported annually, with periodic increases during seasons of elevated tick activity (16). Previous studies have demonstrated active circulation of CCHFV among ixodid ticks, livestock, and human populations in southern Kazakhstan (17). However, epidemiological surveillance in the central regions of the country remains limited, particularly in areas characterized by extensive livestock grazing and seasonal animal movement. As a result, systematic molecular and serological data on CCHFV circulation in livestock and tick populations in these territories remain scarce, limiting understanding of local transmission dynamics and the potential risk of human infection.

In particular, the Karaganda and Ulytau regions are located in Central Kazakhstan and are characterized by extensive pasture-based livestock production systems, seasonal livestock movement, and ecological conditions favorable for the maintenance of ixodid tick populations. In addition, these territories maintain broad geographic connectivity with southern endemic regions of the country, potentially facilitating interregional circulation of CCHFV through movement of animal hosts and tick vectors. Despite these epidemiologically relevant characteristics, systematic molecular and serological surveillance data on CCHFV circulation in livestock and tick populations in these regions remain limited.

Accordingly, these regions represent epidemiologically relevant territories for investigating possible interregional circulation of CCHFV due to their geographic location, livestock movement patterns, and limited availability of systematic surveillance data.

Environmental changes, livestock movement, and the potential expansion of *Hyalomma* tick habitats may contribute to the spread of CCHFV into previously understudied territories, highlighting the need for updated regional surveillance data. In the absence of widely available vaccines or specific antiviral therapy, effective prevention and control of CCHF require integrated One Health approaches combining veterinary, epidemiological, and ecological investigations (18). Such multidisciplinary collaboration has already been implemented in Kazakhstan, including joint surveillance and epidemiological studies conducted in the Zhambyl region involving veterinarians, epidemiologists, and entomologists (17).

Against this background, the present study aimed to assess evidence of CCHFV circulation in cattle, small ruminants, and ixodid ticks collected from administrative districts of Central Kazakhstan. In addition, anti-CCHFV IgG antibodies in livestock were evaluated to assess previous exposure to the virus. The study also included virus isolation from RT-qPCR-positive tick samples, amplification and sequencing of the viral S segment, and phylogenetic characterization of the detected isolate.

To our knowledge, this study represents one of the first integrated investigations providing molecular, serological, and virological evidence suggestive of CCHFV circulation in Central Kazakhstan, including virus isolation and genetic characterization of a local isolate. The obtained nucleotide sequence of the viral S segment was deposited in the GenBank database, contributing to the expanding dataset on the genetic diversity of CCHFV and improving understanding of phylogeographic relationships among strains circulating in Central Asia.

## 2 Materials and methods

### 2.1 Study design and sampling area

A targeted cross-sectional epizootiological surveillance study of Crimean-Congo hemorrhagic fever virus (CCHFV) was conducted during the spring and summer months of 2025, corresponding to the period of increased ixodid tick activity in Central Kazakhstan. The study was carried out in the Ulytau and Karaganda regions. These regions were selected because of their central geographic location, seasonal livestock movement routes connecting southern and central Kazakhstan, extensive pasture-based livestock production systems, ecological suitability for ixodid tick populations, and the limited availability of systematic molecular and serological surveillance data on CCHFV circulation in these territories.

The surveyed areas included the Ulytau and Zhanaarka districts and the cities of Zhezkazgan, Satpayev, and Karazhal in the Ulytau region, as well as the Shet and Aktogay districts in the Karaganda region. Sampling sites were selected purposively rather than randomly based on the presence of grazing livestock, reported or observed tick activity, pasture accessibility, and geographic proximity to historically CCHFV-endemic regions of southern Kazakhstan. The surveyed localities are characterized by extensive pasture use, steppe and semi-arid landscapes, and seasonal environmental conditions favorable for livestock-tick interactions and maintenance of ixodid tick populations.

Accordingly, the study was designed to assess evidence of CCHFV circulation in ecologically relevant livestock-associated environments rather than to estimate population-level prevalence across the entire regions.

**Figure 1:**
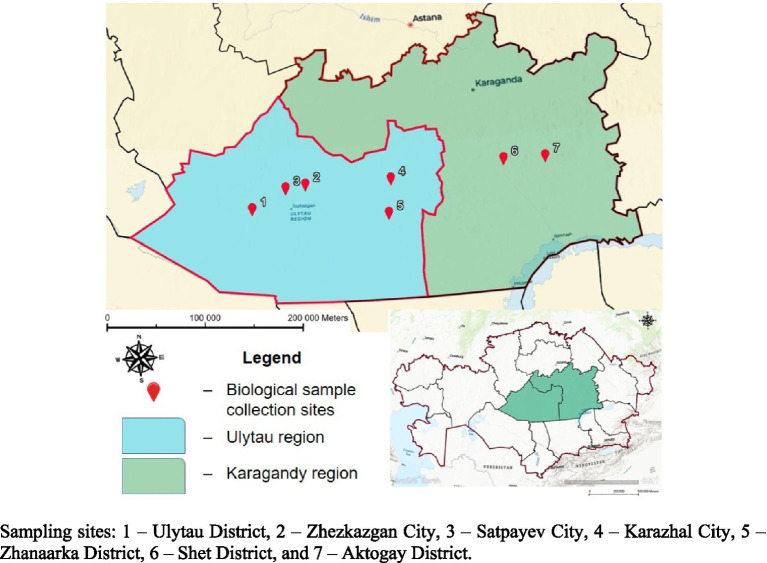
Geographic distribution of sampling sites in Central Kazakhstan. Sampling sites: 1 – Ulytau District, 2 – Zhezkazgan City, 3 – Satpayev City, 4 – Karazhal City, 5 – Zhanaarka District, 6 – Shet District, and 7 – Aktogay District.

Figure 1 shows the geographic distribution of the sampling sites included in the study area.

In each of the seven surveyed localities, samples were collected from 50 cattle and 50 small ruminants, resulting in a total of 350 cattle and 350 small ruminants. Whole blood and serum samples were obtained from the same animals by jugular venipuncture using sterile vacuum collection systems. K2 EDTA tubes (Avatube Ecopharm, Shymkent, Kazakhstan) were used for whole blood collection, whereas serum separator tubes containing clot activator and gel (Avatube Ecopharm, Shymkent, Kazakhstan) were used for serum collection.

Livestock sampling was conducted during field visits to privately owned households and small farms located within the study districts. Within each visited household or farm, animals meeting the inclusion criteria were identified, and cattle and small ruminants were randomly selected from the available livestock population. Animals were included if they belonged to the target species groups and could be safely restrained for sampling with owner consent. Information regarding animal species, locality, sex, and age group distribution is provided in (Supplementary Tables S1, S2).

Simultaneously, a total of 127 ixodid ticks were collected from pastures and directly from animals using flagging, manual removal from host animals, and direct hand collection methods. For each specimen, the collection site, host animal, tick species, developmental stage, and collection date were recorded. Tick species identification and developmental stage determination were performed based on morphological characteristics under a stereomicroscope using standard taxonomic keys (19–21). Ticks were subsequently grouped into pools according to collection site, host species, tick species, and developmental stage. Detailed information on pool composition and size is provided in Supplementary material 1.

All samples were transported to the laboratory under cold-chain conditions and stored at −20 °C until molecular and serological analyses were performed.

### 2.2 RNA extraction

Viral RNA was extracted from livestock blood samples and tick homogenates using the QIAamp Viral RNA Mini Kit (QIAGEN, Hilden, Germany) according to the manufacturer’s instructions. Tick pools were mechanically homogenized in sterile phosphate-buffered saline (PBS) using disposable sterile pestles. Homogenates were clarified by centrifugation, and the resulting supernatants were subsequently used for RNA extraction and virus isolation procedures.

All procedures were performed in a laminar flow cabinet under appropriate biosafety conditions, with precautions taken to minimize RNA degradation and prevent cross-contamination between samples. Extracted RNA was used directly for reverse transcription polymerase chain reaction (RT-PCR) assays targeting fragments of the CCHFV genome.

For sequencing analysis, viral nucleic acids were additionally extracted from tick homogenates using the Quick-RNA Miniprep Plus Kit (Zymo Research, Irvine, CA, United States) following the manufacturer’s recommendations. RNA concentration was measured using the Qubit RNA HS Assay Kit (Life Technologies, Carlsbad, CA, United States) on a Qubit 2.0 fluorometer (Life Technologies, Carlsbad, CA, United States).

Negative extraction controls were included throughout the extraction procedures to monitor potential contamination.

### 2.3 Molecular and serological diagnostics

#### 2.3.1 Molecular detection

Detection of CCHFV RNA was performed using real-time reverse transcription polymerase chain reaction (RT-qPCR) with the RealStar® CCHFV RT-PCR Kit 1.0 (Altona Diagnostics, Germany) according to the manufacturer’s instructions. RT-qPCR assays were carried out on a Rotor-Gene Q 6plex real-time PCR system (QIAGEN, Hilden, Germany) with fluorescence detection. Amplification signals were recorded in the FAM channel for specific detection of CCHFV RNA and in the JOE channel for internal reaction control.

Results were interpreted based on threshold cycle (Ct) values according to the criteria recommended by the manufacturer. Positive and negative controls supplied with the kit were included in each analytical run to ensure assay validity. Samples showing valid amplification in the FAM channel together with an appropriately functioning internal control were considered positive for CCHFV RNA.

#### 2.3.2 Serological detection

Anti-CCHFV IgG antibodies were detected using an enzyme-linked immunosorbent assay (ELISA) with the ID Screen® CCHF Double Antigen Multi-species ELISA kit (IDvet, Grabels, France) according to the manufacturer’s instructions.

Optical density (OD) values were measured at 450 nm using a MultiScan Sky High microplate reader. Results were expressed as sample-to-positive (S/P) percentages. Samples with S/*p* values ≤30% were considered negative, whereas samples with S/p values >30% were interpreted as positive for anti-CCHFV IgG antibodies.

All serum samples were tested individually. Positive and negative controls provided with the kit were included in each analytical run to ensure assay validity.

### 2.4 Amplification and sequencing of the S segment

Specific primers targeting the S segment of the CCHFV genome were designed based on multiple sequence alignment of reference nucleotide sequences using the ClustalW algorithm implemented in MEGA v.12. Primer synthesis was performed at the shared core facility of the Research Institute for Biological Safety Problems (RIBSP).

Reverse transcription polymerase chain reaction (RT-PCR) was performed using the One-Step RT-PCR Kit (QIAGEN, Germany) on a Mastercycler X50s thermocycler (Eppendorf, Germany). Reaction conditions included reverse transcription at 50 °C for 30 min, initial denaturation at 95 °C for 15 min, followed by 35 amplification cycles consisting of denaturation at 94 °C for 20 s, annealing at 55 °C for 30 s, and extension at 72 °C for 1 min, with a final extension step at 72 °C for 7 min. Each 50 μL reaction mixture contained 10 μL of 5 × reaction buffer, 2 μL of 10 mM dNTP mix, 800 nM of each primer, 2 μL of enzyme mix, 3 μL of viral RNA template, and nuclease-free water to the final reaction volume.

Amplification products were analyzed by electrophoresis in 2% agarose gel prepared in TAE buffer containing SYBR Safe DNA Gel Stain (Invitrogen, United States). Amplicons were visualized using an iBright CL1500 gel documentation system (Thermo Fisher Scientific, United States). A 1 kb Plus DNA Ladder (Thermo Fisher Scientific, USA) was used as a molecular size marker.

Purified amplicons were sequenced using the Sanger method with the BigDye Terminator v3.1 Cycle Sequencing Kit (Applied Biosystems, Foster City, CA, United States) on a 3,130 XL Genetic Analyzer. The obtained nucleotide sequences were processed and assembled using Sequencher v.5.4 (Gene Codes Corporation, Ann Arbor, MI, United States). Preliminary strain identification and assessment of genetic relatedness were performed using BLASTN searches against the NCBI nucleotide database.

### 2.5 Phylogenetic analysis

Multiple sequence alignment of the obtained nucleotide sequences with 75 reference CCHFV isolates retrieved from the GenBank database was performed using MEGA v.12.

Reference sequences were selected to represent the major phylogenetic lineages of CCHFV, with particular emphasis on strains previously reported from Kazakhstan, neighboring Central Asian countries, and representative global genotypes. Poorly aligned terminal regions were trimmed prior to phylogenetic reconstruction to improve alignment quality.

Phylogenetic analysis was performed using the maximum likelihood (ML) method based on the Tamura-Nei substitution model (22) with 1,000 bootstrap replicates to assess branch support. The nucleotide sequence generated in the present study was deposited in the GenBank database.

### 2.6 Virus isolation and cultivation

Virus isolation was performed using the following cell cultures: the continuous monolayer-suspension subline of baby hamster kidney cells BHK-21/13 (ATCC CCL-10™), the CV-1 cell line derived from African green monkey kidney (ATCC CCL-70™), the Vero cell line (ATCC CRL-1586™), and the MARC-145 cell line derived from embryonic African green monkey kidney.

All cell cultures were obtained from the Cell Biotechnology Laboratory of the Research Institute for Biological Safety Problems (RIBSP) and maintained in 25 cm^2^ culture flasks containing Dulbecco’s Modified Eagle Medium (DMEM) supplemented with 10% fetal bovine serum (Capricorn Scientific, Ebsdorfergrund, Germany).

RT-qPCR-positive tick homogenate supernatants were used as inocula for virus isolation. Prior to inoculation, tick homogenates were clarified by centrifugation at 3,000 × g for 10 min to remove coarse particulate material and cellular debris. The resulting supernatants were subsequently filtered through sterile 0.22 μm membrane filters. Virus-containing suspensions (0.1 mL per flask) were inoculated onto cell monolayers and allowed to adsorb for 1 h at 37 °C. Following adsorption, the monolayers were washed one to two times with phosphate-buffered saline (PBS), after which infected cultures were incubated for up to 5 days at 37 °C in a humidified atmosphere containing 5% CO₂ in maintenance medium supplemented with 2% fetal bovine serum and L-glutamine (final concentration 6 mM).

Cell cultures were monitored daily by light microscopy for the development of cytopathic effects (CPE). Upon observation of pronounced CPE, culture supernatants were collected, clarified by centrifugation to remove cellular debris, and subsequently used for RT-qPCR confirmation and additional virus passages.

### 2.7 Bioethics

All procedures involving animal sampling were conducted in accordance with the regulations of the Ministry of Agriculture of the Republic of Kazakhstan and were approved by the Committee for Veterinary Control and Supervision under Protocol No. 5 dated 21 November 2023 issued by the Research Institute for Biological Safety Problems (RIBSP).

Animal sampling procedures were performed by trained veterinary personnel using standard handling practices to minimize animal stress and discomfort during sample collection.

All CCHFV-related laboratory work was conducted at the Research Institute for Biological Safety Problems (RIBSP), Kazakhstan, in a BSL-3 laboratory in accordance with national and institutional biosafety regulations. Personnel involved in CCHFV-related laboratory work were trained, certified, and officially authorized to handle biological materials under BSL-3 containment conditions. All procedures were performed using appropriate PPE and institutional containment measures.

### 2.8 Statistical analysis

Statistical analyses were performed using GraphPad Prism v.9.5 (GraphPad Software, United States). Descriptive statistics were used to summarize the study data. Results were expressed as absolute numbers and percentages. The proportions of CCHFV RNA-positive samples and anti-CCHFV IgG-seropositive animals were calculated for the overall sample set and, where applicable, according to animal species, sampling locality, and age group.

Because the study was designed as targeted surveillance in ecologically relevant areas, statistical analyses were intended to provide descriptive assessment of detected findings rather than population-level prevalence estimates for the entire regions.

Geographic mapping and spatial visualization of sampling sites were performed using Esri ArcGIS Pro version 2.2.

## 3 Results

### 3.1 Tick species composition

Morphological identification of ixodid ticks was performed based on key diagnostic characteristics, including the structure of the capitulum, scutum morphology, body coloration, festoons, and, where applicable, male genital structures. A total of 127 tick specimens were collected and identified from surveyed localities in the Ulytau and Karaganda regions (Table 1).

**Table 1:** Morphological identification of tick species and sex distribution (*n* = 127).

Tick species	Total (n)	%	Females (n)	Males (n)
*Hyalomma marginatum*	63	49.6	44	19
*Dermacentor marginatus*	26	20.5	17	9
*Dermacentor reticulatus*	23	18.1	10	13
*Rhipicephalus sanguineus*	10	7.9	4	6
*Ixodes ricinus (nymphs)*	5	3.9	3	2
Total	127	100	78	49

Five ixodid tick species were detected in the examined material: *Hyalomma marginatum, Dermacentor marginatus, Dermacentor reticulatus, Rhipicephalus sanguineus,* and *Ixodes ricinus*. The dominant species was *H. marginatum*, which accounted for 49.6% (63/127) of all collected ticks. The second and third most frequently detected species were *D. marginatus* (20.5%; 26/127) and *D. reticulatus* (18.1%; 23/127), respectively. Less frequently encountered species included *R. sanguineus* (7.9%; 10/127) and *I. ricinus* (3.9%; 5/127), the latter being represented exclusively by nymphs. Thus, nearly half of all identified ticks belonged to the genus *Hyalomma*, whereas the remaining half was distributed among four other ixodid species.

Analysis of sex distribution showed a predominance of females in the overall tick collection. Of the 127 examined specimens, 78 (61.4%) were females and 49 (38.6%) were males. Female predominance was observed particularly in *H. marginatum* (44 females vs. 19 males) and *D. marginatus* (17 females vs. 9 males), whereas *D. reticulatus* showed a slight predominance of males (13 males vs. 10 females). In *R. sanguineus*, males also slightly predominated (6 males vs. 4 females). Although the number of *I. ricinus* specimens was low, this species was likewise represented mainly by immature stages. These findings indicate that the collected material included both abundant adult vectors and a smaller proportion of less frequently encountered taxa.

The body length of the examined ticks ranged from approximately 3.2 mm in nymphs to 14 mm in engorged females. Adult females of *H. marginatum* measured approximately 5.0–6.5 mm in the unfed state, whereas adult *Dermacentor* spp. ticks were smaller, ranging from 4.6–5.4 mm in *D. marginatus* and 3.8–4.2 mm in *D. reticulatus*.

Overall, the tick fauna detected in the study area was characterized by predominance of *H. marginatum*, accompanied by substantial representation of *Dermacentor* spp. and lower proportions of *R. sanguineus* and *I. ricinus*. This species composition provided the basis for subsequent molecular screening of tick pools for CCHFV RNA.

### 3.2 Detection of viral RNA and CCHFV-specific antibodies

#### 3.2.1 Molecular detection of CCHFV RNA

A total of 700 whole blood samples obtained from livestock, including 350 samples from cattle and 350 from small ruminants, were analyzed for the presence of CCHFV RNA using RT-qPCR. No viral RNA was detected in any of the examined livestock blood samples (0/700).

**Figure 2:**
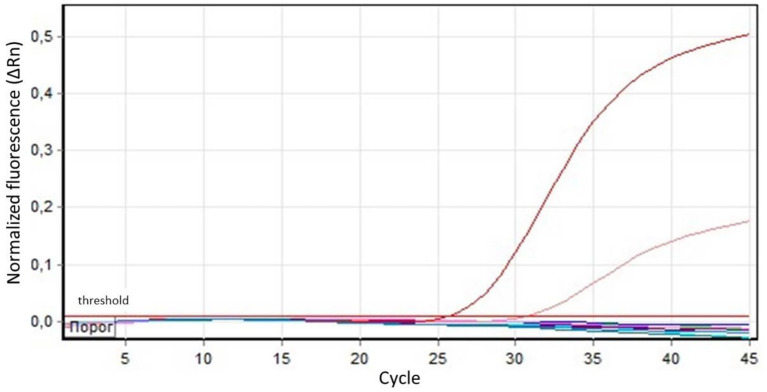
Real-time RT-qPCR detection of CCHFV RNA in the positive tick pool obtained from the Mibulak rural district (Ct = 30.77; internal control Ct = 25.73).

Among tick samples, one of eight tested pools (12.5%) yielded a positive RT-qPCR result for CCHFV RNA. The positive pool originated from the Mibulak rural district of the Ulytau region (48.830480 N, 68.412196 E) and produced a Ct value of 30.77, whereas the internal control Ct value was 25.73 (Figure 2). Based on one RT-qPCR-positive tick pool among 127 tested ticks, the minimum infection rate (MIR), calculated under the assumption that only one tick in the positive pool was infected, was 7.87 per 1,000 ticks, equivalent to 0.79%.

All remaining tick pools tested negative for CCHFV RNA. These findings indicate detection of CCHFV RNA in ixodid tick material collected in the Ulytau region, whereas no evidence of detectable viremia was identified in the examined livestock at the time of sampling.

#### 3.2.2 Serological testing

To assess previous exposure to CCHFV, a total of 700 serum samples were analyzed, including 350 samples from cattle and 350 from small ruminants. Anti-CCHFV IgG antibodies were detected in 8 of 700 serum samples, corresponding to an overall seropositivity rate of 1.14%. Four of the eight seropositive animals (50.0%) originated from the Mibulak rural district of the Ulytau region.

**Figure 3:**
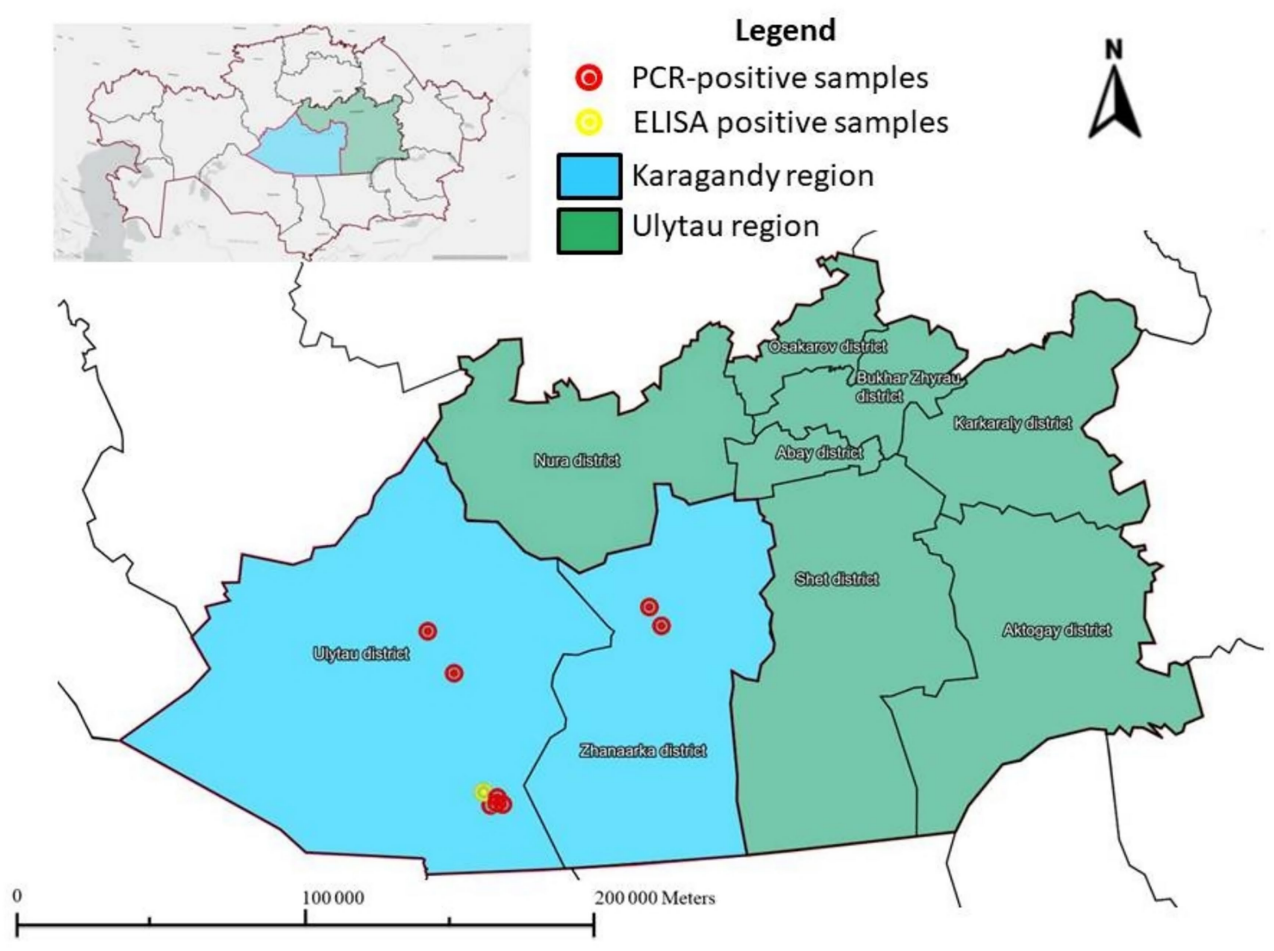
Geographic location of the sampling site in the Ulytau region where both CCHFV RNA-positive tick material and seropositive livestock were identified.

Seropositive animals were identified in both cattle and small ruminants. The geographic distribution of seropositive samples was heterogeneous, with co-occurrence of an RT-qPCR-positive tick pool and seropositive livestock detected in the Mibulak rural district (Figure 3).

Analysis of the age distribution of seropositive animals showed that antibodies to CCHFV were detected both in young animals (up to 2 years of age) and in adults older than 4 years. In small ruminants, seropositivity was predominantly observed in animals aged 3 to 5 years, likely reflecting the cumulative effect of repeated exposure to infected ticks of the genus *Hyalomma* under pasture-based management conditions. At the same time, the detection of seropositive animals in the younger age group (1 year 9 months) indicates ongoing virus circulation in natural foci and suggests relatively early exposure of animals in endemic or potentially endemic areas.

Among cattle, seropositivity was identified in both adult animals (4 years 9 months) and younger individuals (1 year 11 months), indicating that infection may occur during the early stages of life. This may be associated with longer grazing periods, larger pasture areas, and a higher likelihood of contact with tick vectors compared to animals maintained under confined or stall-based conditions.

### 3.3 Virus isolation in cell culture

**Figure 4:**
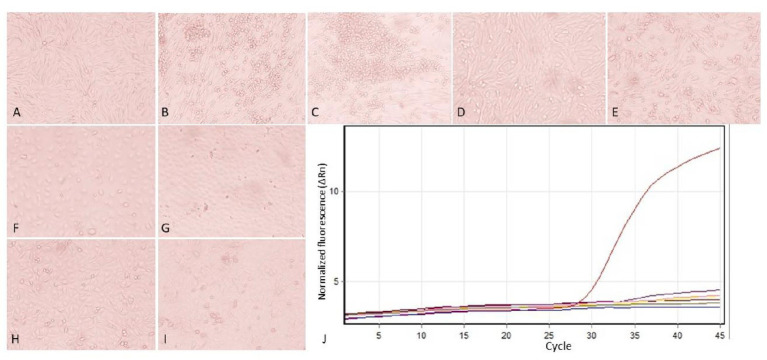
Cytopathic effects observed in different cell cultures following inoculation with CCHFV-positive tick material and RT-qPCR confirmation of viral RNA. **(A)** Uninfected BHK-21/13 cell monolayer; **(B)** CPE in BHK-21/13 cells at 4 days post-infection (dpi); **(C)** CPE in BHK-21/13 cells at 5 dpi; **(D)** Uninfected Vero cell culture; **(E)** CPE in Vero cells at 5 dpi; **(F)** Uninfected MARC-145 cell culture; **(G)** morphological alterations in MARC-145 cells at 5 dpi; **(H)** Uninfected CV-1 cell monolayer; **(I)** morphological alterations in CV-1 cells at 4 dpi; **(J)** RT-qPCR amplification results for virus-containing culture supernatants. Microscopic examination was performed at ×10 magnification.

Virus isolation was performed using monolayers of cell cultures inoculated with material obtained from the RT-qPCR-positive tick pool collected in the Mibulak rural district of the Ulytau region. A pronounced cytopathic effect (CPE) was first observed in BHK-21/13 and Vero cells during the second passage at 4–5 days post-infection. In total, three sequential passages, including two blind passages, were performed. The observed CPE was characterized by cell rounding, detachment, and focal disruption of the cell monolayer, consistent with virus-associated cytopathic changes. In contrast, CV-1 and MARC-145 cultures demonstrated only limited morphological alterations during the observation period, including after blind passaging. Microscopic examination was performed at ×10 magnification (Figures 4A–I).

After 5 days of incubation, culture supernatants were analyzed by RT-qPCR. CCHFV RNA was detected in supernatants obtained from BHK-21/13 and Vero cultures, whereas no viral RNA signal was detected in CV-1 or MARC-145 cultures. RT-qPCR analysis of positive culture supernatants yielded Ct values of 36.01 and 39.92, respectively. The positive control yielded a Ct value of 26.13, whereas no amplification was detected in the negative control.

Taken together, these findings demonstrate detectable CCHFV RNA in BHK-21/13 and Vero culture supernatants following inoculation and passaging, whereas no RT-qPCR-confirmed viral RNA signal was detected in CV-1 or MARC-145 cultures under the experimental conditions used.

After virus isolation and characterization, the CCHF/2025/Mibulak/KZ isolate was deposited in the Collection of Microorganisms at the Research Institute for Biological Safety Problems. All remaining biological materials were destroyed in accordance with institutional and national biosafety regulations.

### 3.4 Molecular genetic characterization of the CCHFV isolate

To characterize the detected CCHFV isolate, the nucleoprotein (NP) gene located within the S segment of the viral genome was selected for amplification and sequencing analysis. A total of 75 reference nucleotide sequences representing the CCHFV S segment were retrieved from the GenBank database and used for primer design and comparative sequence analysis. Multiple sequence alignment was performed using MEGA v.12 to identify conserved regions suitable for primer development.

Three overlapping primer pairs were designed to amplify the target region of the S segment and ensure near-complete sequence coverage (Table 2). Primer design was based on conserved regions identified during sequence alignment analysis. The following parameters were considered during primer development: primer length, melting temperature (Tm), GC content, predicted specificity, self-complementarity and cross-complementarity, absence of extended homopolymeric regions, and potential formation of secondary structures and primer dimers. Overlapping amplicon design was used to improve sequence continuity across the analyzed S-segment region.

**Table 2:** Characteristics of primers designed for amplification and sequencing of the CCHFV S segment.

Primer name	Sequence (5’–3’)	Tm (°C)	GC (%)	Product size (bp)
cchf-bf1	CTC AAA GAA ACA CGT GCC GC	55	55	562
cchf-br1	AAC ATT TCT TTG ACR GAC AT	50	35	
cchf-bf2	TTC CGT GTC AAT GCA AAT AC	50	40	714
cchf-br2	GGT GCA TGT AGA TCC TGT T	50	47	
cchf-bf3	TCC CAA CTG TCT CAC AGT T	50	47	654
cchf-br3	CTC AAA GAT ATC GTT GCC GC	53	50	

The designed primers were evaluated in silico for sequence specificity using the BLAST tool available through the NCBI platform. Primer synthesis was performed at the core facility of the Research Institute for Biological Safety Problems. Working primer solutions were prepared at a final concentration of 20 pmol/μL.

**Figure 5:**
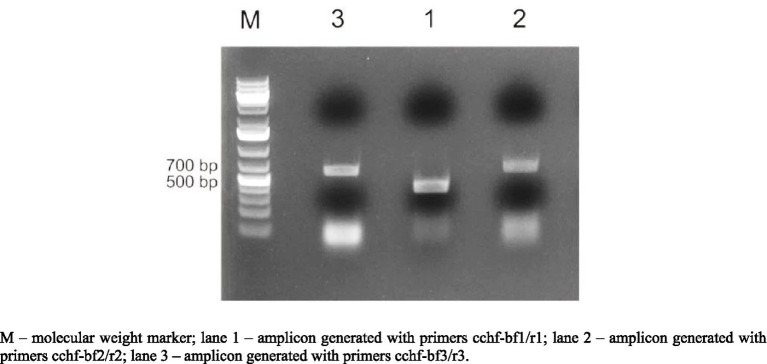
Agarose gel electrophoresis of RT-PCR products corresponding to the CCHFV S segment amplified using sequencing primers. M – molecular weight marker; lane 1 – amplicon generated with primers cchf-bf1/r1; lane 2 – amplicon generated with primers cchf-bf2/r2; lane 3 – amplicon generated with primers cchf-bf3/r3.

RT-PCR amplification using the designed primer pairs, followed by agarose gel electrophoresis, resulted in successful amplification of the expected CCHFV-specific fragments from isolate CCHF/2025/Mibulak/KZ (Figure 5).

Amplified PCR products were subsequently sequenced using the Sanger method. Sequence assembly generated a 1,651 bp fragment corresponding to the CCHFV S segment. The obtained nucleotide sequence was deposited in the GenBank database under accession number PX210841. Annotation of the assembled sequence demonstrated that the analyzed fragment encodes a nucleoprotein (NP) consisting of 482 amino acids (Supplementary material 2).

These findings confirmed successful amplification, sequencing, and assembly of the S-segment fragment of isolate CCHF/2025/Mibulak/KZ.

### 3.5 Phylogenetic analysis

**Figure 6:**
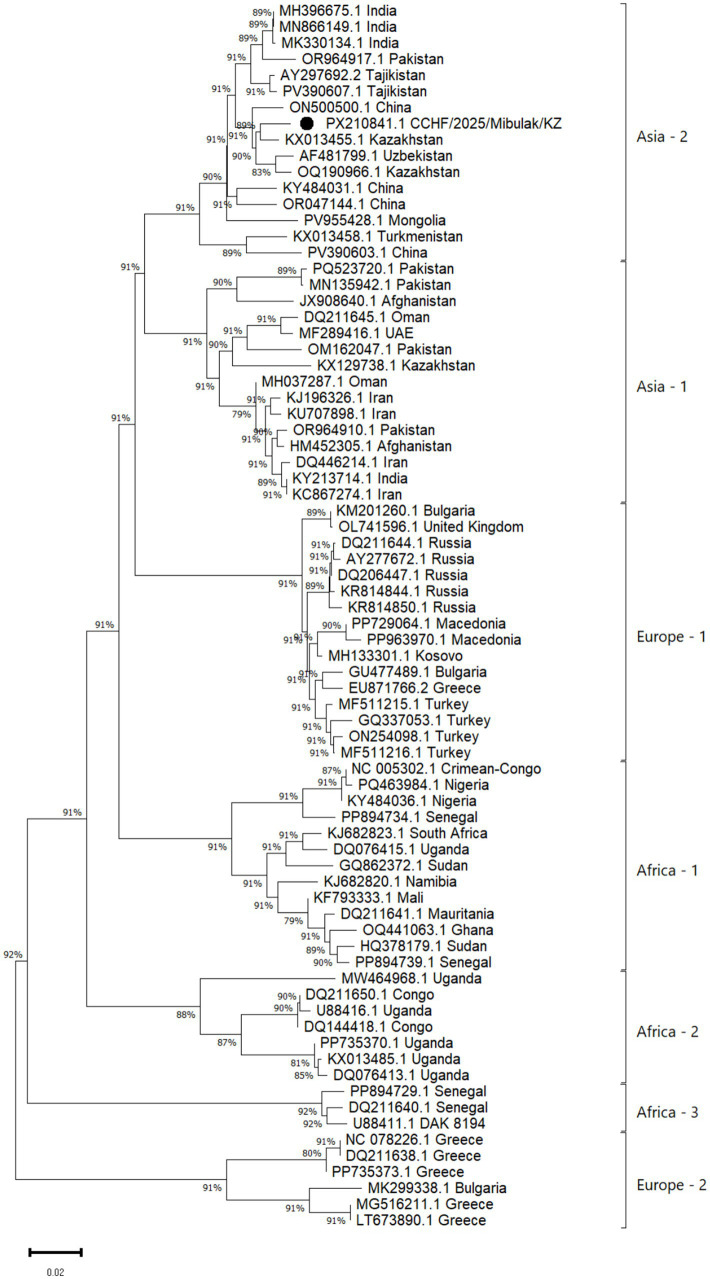
Phylogenetic analysis of the S segment of CCHFV isolate CCHF/2025/Mibulak/KZ.

For phylogenetic analysis, the obtained S-segment nucleotide sequence was compared with reference CCHFV isolates retrieved from public databases. Multiple sequence alignment and phylogenetic tree reconstruction were performed using the maximum likelihood (ML) method (23). Branch lengths were expressed as the number of nucleotide substitutions per site, and bootstrap support values are indicated next to the corresponding nodes. The isolate identified in the present study is marked with a black circle in the phylogenetic tree (Figure 6).

Phylogenetic analysis demonstrated that the S segment of isolate CCHF/2025/Mibulak/KZ (GenBank accession no. PX210841) clustered within the Asia-2 genotype with strong bootstrap support (BS = 91%), together with reference strains previously reported from China, Tajikistan, India, Turkmenistan, Uzbekistan, Pakistan, Mongolia, and Kazakhstan.

The studied isolate formed a distinct monophyletic cluster (BS = 89%) with reference sequences ON500500.1 (JMN01/CCHFV/CHINA/2021) from China, KX013455.1 (K128_76) and OQ190966 (KAZ/18/2018) from Kazakhstan, and AF481799.1 (Uzbek/TI10145) from Uzbekistan.

Taken together, these molecular and phylogenetic findings indicate that isolate CCHF/2025/Mibulak/KZ belongs to the Asia-2 lineage and is genetically related to previously reported CCHFV strains circulating in Central Asia and neighboring regions.

## 4 Discussion

Crimean-Congo hemorrhagic fever (CCHF) remains one of the most important tick-borne zoonotic infections because of its wide geographic distribution and potential for severe outbreaks in humans. Globally, expansion of *Hyalomma* tick populations has been associated with climate change, intensification of livestock production, animal trade, and migratory bird movements, all of which may contribute to the emergence of new CCHF foci in previously non-endemic areas (24, 25).

In Kazakhstan, CCHF is considered endemic, particularly in the southern regions, including Zhambyl, Turkestan, Kyzylorda, and the city of Shymkent, where sustained circulation of CCHFV among tick populations and animal hosts has been documented together with recurrent human cases (17, 26). Recent serological investigations have additionally demonstrated the presence of CCHFV-specific antibodies in livestock from multiple regions of the country, suggesting that virus circulation may extend beyond historically recognized endemic territories (27).

National surveillance data for 2024 indicate that, despite a reduction in reported clinical cases compared with previous years, the risk of CCHF transmission remains substantial in endemic regions of Kazakhstan, particularly during periods of increased tick activity (28).

The present study provides molecular, serological, and virological evidence suggestive of CCHFV circulation in Central Kazakhstan, including detection of viral RNA in ixodid ticks, seropositivity in livestock, virus isolation in cell culture, and phylogenetic characterization of the detected isolate. Taken together, these findings are consistent with possible localized circulation of CCHFV in the Ulytau region beyond the historically recognized southern endemic belt of Kazakhstan.

Our molecular findings are particularly relevant because CCHFV RNA was detected in a tick pool collected in the Mibulak rural district, and virus-associated cytopathic effects together with RT-qPCR-positive culture supernatants were subsequently observed in susceptible cell cultures. However, the molecular evidence was limited to a single RT-qPCR-positive tick pool. Therefore, these findings should be interpreted as suggestive of localized CCHFV activity rather than as definitive evidence of widespread or established virus circulation across Central Kazakhstan. Although only a single tick pool tested positive, these findings support the biological relevance of the detected viral material.

Serological findings complemented the molecular results obtained in the present study. Anti-CCHFV IgG antibodies were detected in livestock from Central Kazakhstan, indicating previous exposure to the virus. Detection of seropositive animals in the absence of clinical signs is consistent with the predominantly subclinical nature of CCHFV infection in cattle and small ruminants reported in previous studies. Seropositive animals were identified in both cattle and small ruminants, with half of all seropositive samples originating from the Mibulak rural district, where RT-qPCR-positive tick material was also detected. The co-occurrence of seropositive livestock and RT-qPCR-positive tick material within the same locality may support the possibility of localized virus circulation in the surveyed area. Nevertheless, the overall livestock seropositivity rate was low (1.14%), which may reflect sporadic or localized exposure to infected ticks rather than established endemic circulation. Therefore, these serological findings should be considered supportive evidence of possible CCHFV exposure in the surveyed localities, but not definitive evidence of endemicity.

The geographic distribution of seropositive animals additionally suggests that CCHFV exposure in Central Kazakhstan may not be limited to a single locality. Seropositive livestock were identified in several surveyed areas of the Ulytau region, including Mibulak, Zhezkazgan, Satpayev, and Karazhal. Although these findings do not by themselves confirm the presence of stable natural foci, they are consistent with geographically heterogeneous virus exposure in this part of the country. Similar observations have been reported in previous studies demonstrating that livestock seropositivity may serve as an indicator of silent CCHFV circulation even in the absence of recognized human cases or clinically apparent disease in animals.

The predominance of *Hyalomma* ticks in the collected material is also relevant for interpreting the present findings. Species of this genus are recognized as major vectors of CCHFV and are capable of maintaining the virus through both transstadial and transovarial transmission. Therefore, the detection of viral RNA in tick material collected from an area where *Hyalomma* ticks are abundant is epidemiologically plausible and consistent with the established ecology of the virus. However, because ticks were tested as pooled samples, the true infection prevalence among individual ticks could not be determined, and species-specific infection rates should be interpreted with caution. Based on one RT-qPCR-positive tick pool among 127 tested ticks, the calculated minimum infection rate (MIR) was 7.87 per 1,000 ticks, equivalent to 0.79%; however, this value represents only a minimum estimate based on the assumption that a single tick in the positive pool was infected. Accordingly, our results should be interpreted cautiously, as the current study was not designed to estimate individual tick infection prevalence or species-specific infection rates.

Comparison with previously published data from other regions of Kazakhstan further supports the relevance of the present findings. Earlier studies have reported substantial livestock seropositivity in southern endemic regions, including Zhambyl, Turkestan, and Kyzylorda, as well as detection of CCHFV-positive ticks in both recognized endemic areas and territories not previously classified as endemic (23, 27, 29–31). Against this background, the present study provides additional evidence suggestive of CCHFV circulation in Central Kazakhstan using an integrated serological, molecular, virological, and phylogenetic approach.

Phylogenetic analysis demonstrated that isolate CCHF/2025/Mibulak/KZ belongs to the Asia-2 genotype and clusters with previously reported strains from China, Kazakhstan, and Uzbekistan. These findings indicate that the detected isolate is genetically related to CCHFV lineages circulating in Central Asia. The observed clustering pattern may reflect regional epidemiological connectivity associated with livestock movement, ecological continuity, and circulation of infected tick vectors and animal hosts across neighboring territories. However, because phylogenetic characterization in the present study was limited to the S segment, interpretation of evolutionary relationships should be made cautiously until additional genomic data become available.

The high Ct values observed in culture-positive materials are consistent with the natural origin of the material and are typical for CCHFV isolates obtained from ticks. Importantly, despite the low RNA load, the virus remained biologically active and capable of replication in susceptible cell cultures. This finding emphasizes that even samples with low detectable RNA concentrations may yield viable virus and should not be dismissed as epidemiologically uninformative.

Several limitations of the present study should be acknowledged. First, surveillance was conducted during a single transmission season and targeted ecologically relevant areas characterized by expected tick activity and conditions favorable for CCHFV circulation. Therefore, the findings should be interpreted as evidence suggestive of CCHFV exposure and possible circulation within the surveyed localities rather than as definitive prevalence estimates for the entire Ulytau and Karaganda regions. Second, pooled tick testing precluded estimation of infection prevalence among individual ticks and determination of the exact number of infected specimens within the positive pool, and calculation of species-specific infection rates. Although the calculated MIR was 7.87 per 1,000 ticks, equivalent to 0.79%, this value should be interpreted only as a minimum estimate and not as a definitive measure of true tick infection prevalence. Third, genomic characterization was limited to analysis of the S segment, whereas full-genome sequencing would provide more comprehensive insight into genetic diversity, reassortment events, and phylogeographic relationships of circulating strains. Finally, the relatively low number of positive samples limited broader quantitative interpretation regarding the extent of virus circulation in the study area. Nevertheless, the combined serological, molecular, virological, and phylogenetic findings support the biological and epidemiological relevance of the detected CCHFV-associated material in Central Kazakhstan.

Overall, the findings of this study should be interpreted within the context of targeted seasonal surveillance rather than as a definitive assessment of regional CCHFV prevalence. The detection of CCHFV-associated markers in livestock and ticks from Central Kazakhstan indicates that territories located outside the traditionally recognized southern endemic belt may require closer epidemiological attention. Future surveillance should include repeated seasonal sampling, larger numbers of individually tested ticks, ecological characterization of sampling sites, and full-genome sequencing of detected strains. Such data would help clarify whether the observed signal reflects sporadic introduction, transient virus activity, or the formation of stable local transmission cycles in central Kazakhstan.

## 5 Conclusion

The present study provides molecular, serological, and virological evidence suggestive of possible localized CCHFV activity in Central Kazakhstan beyond the historically recognized southern endemic regions of the country. The combined detection of anti-CCHFV IgG antibodies in livestock, CCHFV RNA in ixodid tick material, and virus isolation and genetic characterization of isolate CCHF/2025/Mibulak/KZ support the epidemiological relevance of the detected signal in the Ulytau region. However, considering the single RT-qPCR-positive tick pool and the low livestock seropositivity rate, these findings should be interpreted as evidence of localized exposure and possible virus activity rather than as definitive evidence of widespread or established endemic circulation. Phylogenetic analysis demonstrated that the detected isolate belongs to the Asia-2 genotype and is genetically related to previously reported Central Asian strains. Taken together, these findings highlight the need for continued integrated surveillance in Central Kazakhstan within a One Health framework, with particular attention to livestock-associated exposure, tick monitoring, and early detection of potential CCHFV transmission risks.

## Data Availability

The original contributions presented in this study are publicly available. The nucleotide sequence generated in this study is available in the GenBank database under accession number PX210841.

## References

[1] Sheek-Hussein M Zewude A Abdullahi AS Neyadi JA Osman B Hassen AA . Crimean–Congo hemorrhagic fever virus infections in slaughtered camels and abattoir workers in the United Arab Emirates. Transbound Emerg Dis. (2025) 2025:3409106. doi: 10.1155/tbed/3409106, 40376207 PMC12081147

[2] Hawman DW Feldmann H. Crimean-Congo haemorrhagic fever virus. Nat Rev Microbiol. (2023) 21:463–77. doi: 10.1038/s41579-023-00871-9, 36918725 PMC10013989

[3] Kong Y Yan C Liu D Jiang L Zhang G He B . Phylogenetic analysis of Crimean-Congo hemorrhagic fever virus in inner Mongolia, China. Ticks Tick Borne Dis. (2021) 13:101856. doi: 10.1016/j.ttbdis.2021.101856, 34763306

[4] WHO. *Crimean-Congo Haemorrhagic Fever, World Health Organization (WHO) Report*. (2025). Available online at: https://www.who.int/health-topics/crimean-congo-haemorrhagic-fever (Accessed April 12, 2025).

[5] Frank MG Weaver G Raabe V. Crimean-Congo hemorrhagic fever virus for clinicians—diagnosis, clinical management, and therapeutics. Emerg Infect Dis. (2024) 30:648. doi: 10.3201/eid3005.231648 PMC11060459 38666553

[6] Kaya S Elaldi N Kubar A Gursoy N Yilmaz M Karakus G . Sequential determination of serum viral titers, virus-specific IgG antibodies, and TNF-α, IL-6, IL-10, and IFN-γ levels in patients with Crimean-Congo hemorrhagic fever. BMC Infect Dis. (2014) 14:416. doi: 10.1186/1471-2334-14-416, 25066751 PMC4133611

[7] Wang Y Dutta S Karlberg H Devignot S Weber F Hao Q . Structure of Crimean-Congo hemorrhagic fever virus nucleoprotein: Superhelical Homo-oligomers and the role of Caspase-3 cleavage. J Virol. (2012) 86:12294–303. doi: 10.1128/JVI.01627-12, 22951837 PMC3486442

[8] Golden JW Fitzpatrick CJ Suschak JJ Clements TL Ricks KM Sanchez-Lockhart M . Induced protection from a CCHFV-M DNA vaccine requires CD8+ T cells. Virus Res. (2023) 334:199173. doi: 10.1016/j.virusres.2023.199173, 37459918 PMC10388194

[9] Frank MG Weaver G Raabe V. Crimean-Congo hemorrhagic fever virus for clinicians—virology, pathogenesis, and pathology. Emerg Infect Dis. (2024) 30:847–53. doi: 10.3201/eid3005.231646, 38666566 PMC11060449

[10] Schulz A Barry Y Stoek F Ba A Schulz J Haki ML . Crimean-Congo hemorrhagic fever virus antibody prevalence in Mauritanian livestock (cattle, goats, sheep and camels) is stratified by the animal’s age. PLoS Negl Trop Dis. (2021) 15:228. doi: 10.1371/journal.pntd.0009228, 33844691 PMC8081336

[11] Bernard C Apolloni A Grosbois V Peyraud A Saengram P Jori F . First detection of Crimean Congo hemorrhagic fever antibodies in cattle and wildlife of southern continental France: investigation of explanatory factors. PLoS One. (2025) 20:1875. doi: 10.1371/journal.pone.0331875, 40991540 PMC12459774

[12] Gilbride C Saunders J Sharpe H Maze EA Limon G Ludi AB . The integration of human and veterinary studies for better understanding and Management of Crimean-Congo Haemorrhagic Fever. Front Immunol. (2021) 12:636. doi: 10.3389/fimmu.2021.629636, 33815379 PMC8012513

[13] Gazezova S Gabdullina M Ayapova G Nabirova D Waltenburg M Smagul M . Outbreak of Crimean-Congo hemorrhagic fever in Kyzylorda region, Kazakhstan, march–July 2022. Front Public Health. (2022) 13:261. doi: 10.3389/fpubh.2025.1519261, 40308926 PMC12040912

[14] Goedhals D Bester PA Paweska JT Swanepoel R Burt FJ. Next-generation sequencing of southern African Crimean-Congo haemorrhagic fever virus isolates reveals a high frequency of M segment reassortment. Epidemiol Infect. (2014) 142:1952–62. doi: 10.1017/s0950268814000818, 24786748 PMC9151272

[15] Bente DA Forrester NL Watts DM McAuley AJ Whitehouse CA Bray M. Crimean-Congo hemorrhagic fever: history, epidemiology, pathogenesis, clinical syndrome and genetic diversity. Antivir Res. (2013) 100:159–89. doi: 10.1016/j.antiviral.2013.07.006, 23906741

[16] Berdikulov M Maikhin K Karibayev T Kalkabayev K Kazybay B Nissanova R . Genetic evidence of regional circulation of Crimean-Congo hemorrhagic fever virus in ixodid ticks from southern Kazakhstan. Front Vet Sci. (2025) 12:822. doi: 10.3389/fvets.2025.1623822, 41112156 PMC12532773

[17] CDC. *Understanding Crimean-Congo Hemorrhagic Fever in Kazakhstan*. One Health. (2024). Available online at: https://www.cdc.gov/one-health/php/stories/cchf.html.

[18] Sorvillo TE Rodriguez SE Hudson P Carey M Rodriguez LL Spiropoulou CF . Towards a sustainable one health approach to Crimean–Congo hemorrhagic fever prevention: focus areas and gaps in knowledge. Trop Med Infect Dis. (2020) 5:113. doi: 10.3390/tropicalmed5030113, 32645889 PMC7558268

[19] Filippova NA. Fauna of Russia and Adjacent Countries. Ixodid Ticks of Subfamily Ixodinae (Parasitiformes, Ixodidae). St. Petersburg: Nauka (1997).

[20] Guglielmone AA Robbins RG Apanaskevich DA Petney TN Estrada-Peña A Horak IG. The Hard Ticks of the World. Berlin: Springer (2014).

[21] Apanaskevich DA Horak IG. The genus Hyalomma Koch, 1844: v. re-evaluation of the taxonomic rank of taxa comprising the H. (Euhyalomma) marginatum koch complex of species (Acari: Ixodidae) with redescription of all parasitic stages and notes on biology. Int J Acarol. (2008) 34:13–42. doi: 10.1080/01647950808683704

[22] Tamura K Nei M Kumar S. Prospects for inferring very large phylogenies by using the neighbor-joining method. Proc Natl Acad Sci. (2004) 101:11030–5. doi: 10.1073/pnas.0404206101, 15258291 PMC491989

[23] Saitou N Nei M. The neighbor-joining method: a new method for reconstructing phylogenetic trees. Mol Biol Evol. (1987) 4:406–25. doi: 10.1093/oxfordjournals.molbev.a040454, 3447015

[24] Rodrigues MA Lesiczka P MariaCardoso L Coelho AC. The expanding threat of Crimean-Congo haemorrhagic fever virus: role of migratory birds and climate change as drivers of *Hyalomma* spp. dispersal in Europe. Birds. (2025) 6:31. doi: 10.3390/birds6020031

[25] Celina SS Černý J Samy AM. Mapping the potential distribution of the principal vector of Crimean-Congo haemorrhagic fever virus *Hyalomma marginatum* in the Old World. PLoS Negl Trop Dis. (2023) 17:855. doi: 10.1371/journal.pntd.0010855 PMC10703407 38011221

[26] Sultankulova KT Shynybekova GO Kozhabergenov NS Mukhami NN Chervyakova OV Burashev YD . The prevalence and genetic variants of the CCHF virus circulating among ticks in the southern regions of Kazakhstan. Pathogens (Basel). (2022) 11:841. doi: 10.3390/pathogens11080841, 36014962 PMC9414327

[27] Abuova G Berdaliyeva F Polukchi T Aliyev D Raymkulov G Kulemin M . Seroprevalence of Crimean-Congo hemorrhagic fever virus in the population of Turkestan region. Infez Med. (2024) 32:11. doi: 10.53854/liim-3201-11 PMC10917566 38456018

[28] National Center for Public Health. *Crimean-Congo hemorrhagic fever in Kazakhstan: Incidence Dynamics in 2024 and Preventive Measures*. National Center for Public Health (2025). Available online at: https://hls.kz/ru/archives/46341.

[29] Bryant-Genevier J Bumburidi Y Kazazian L Seffren V Head JR Berezovskiy D . Prevalence of Crimean-Congo hemorrhagic fever virus among livestock and ticks in Zhambyl region, Kazakhstan, 2017. Am J Trop Med Hyg. (2022) 106:1478–85. doi: 10.4269/ajtmh.21-1092, 35378505 PMC9128673

[30] Sultankulova KT Shynybekova GO Issabek AU Mukhami NN Melisbek AM Chervyakova OV . The prevalence of pathogens among ticks collected from livestock in Kazakhstan. Pathogens. (2022) 11:206. doi: 10.3390/pathogens11101206, 36297263 PMC9611691

[31] World Health Organization (WHO). *Crimean-Congo Hemorrhagic Fever*. (2023). Available online at: https://www.who.int/news-room/fact-sheets/detail/crimean-congo-haemorrhagic-fever.

