# Supplementary material for: Integrated surveillance, virus isolation and phylogenetic characterization of Crimean-Congo hemorrhagic fever virus in Central Kazakhstan
Source: Front Vet Sci. 2026 Jul 16;13:1879322. doi: 10.3389/fvets.2026.1879322 (PMC13421902; doi:10.3389/fvets.2026.1879322)
Supplement: Supplementary file 1 [file Table_1.DOCX]

This supplementary file summarizes the sex and age distribution of the examined cattle and small ruminants included in the study population.

**Supplementary Table S1. Sex distribution of the examined animals by species**

| **Species** | **Female, n (%)** | **Male, n (%)** | **Total** |
| --- | --- | --- | --- |
| Cattle | 276 (78.9) | 74 (21.1) | 350 |
| Small ruminants | 305 (87.1) | 45 (12.9) | 350 |

**Supplementary Table S2. Age distribution of the examined animals by species**

| **Species** | **≤1 year, n (%)** | **>1-3 years, n (%)** | **>3-5 years, n (%)** | **>5-7 years, n (%)** | **>7 years, n (%)** | **Total** |
| --- | --- | --- | --- | --- | --- | --- |
| Cattle | 15 (4.3) | 160 (45.7) | 101 (28.9) | 38 (10.9) | 36 (10.3) | 350 |
| Small ruminants | 5 (1.4) | 83 (23.7) | 186 (53.1) | 47 (13.4) | 29 (8.3) | 350 |

Sampling site: Zhezkazgan

GPS: Широта: 47.629058 Долгота: 67.677760

| **№**  **п/п** | Animal species | Sex and age |
| --- | --- | --- |
| 1 | SMALL RUMINANTS | Female 7 г. 1 м. |
| 2 | SMALL RUMINANTS | Female 4 г. 2 м. |
| 3 | SMALL RUMINANTS | Female 1 г. 11 м. |
| 4 | SMALL RUMINANTS | Female 5 г. 4 м. |
| 5 | SMALL RUMINANTS | Female 2 г. 4 м. |
| 6 | SMALL RUMINANTS | Female 2 г. 4 м. |
| 7 | SMALL RUMINANTS | Female 1 г. 11 м. |
| 8 | SMALL RUMINANTS | Female 2 г. 4 м. |
| 9 | SMALL RUMINANTS | Female 1 г. 2 м. |
| 10 | SMALL RUMINANTS | Female 5 г. 3 м. |
| 11 | SMALL RUMINANTS | Female 1 г. 11 м. |
| 12 | SMALL RUMINANTS | Female 2 г. 4 м. |
| 13 | SMALL RUMINANTS | Female 5 г. 3 м. |
| 14 | SMALL RUMINANTS | Female 2 г. 4 м. |
| 15 | SMALL RUMINANTS | Female 4 г. 2 м. |
| 16 | SMALL RUMINANTS | Female 2 г. 0 м. |
| 17 | SMALL RUMINANTS | Female 2 г. 4 м. |
| 18 | SMALL RUMINANTS | Female 2 г. 4 м. |
| 19 | SMALL RUMINANTS | Female 7 г. 1 м. |
| 20 | SMALL RUMINANTS | Female 7 г. 0 м. |
| 21 | SMALL RUMINANTS | Female 2 г. 0 м. |
| 22 | SMALL RUMINANTS | Female 1 г. 2 м. |
| 23 | SMALL RUMINANTS | Female 5 г. 3 м. |
| 24 | SMALL RUMINANTS | Female 2 г. 3 м. |
| 25 | SMALL RUMINANTS | Female 2 г. 0 м. |
| 26 | SMALL RUMINANTS | Male 2 г. 3 м. |
| 27 | SMALL RUMINANTS | Female 2 г. 4 м. |
| 28 | SMALL RUMINANTS | Female 2 г. 0 м. |
| 29 | SMALL RUMINANTS | Male 3 г. 1 м. |
| 30 | SMALL RUMINANTS | Female 2 г. 0 м. |
| 31 | SMALL RUMINANTS | Female 4 г. 2 м. |
| 32 | SMALL RUMINANTS | Female 1 г. 11 м. |
| 33 | SMALL RUMINANTS | Female 2 г. 0 м. |
| 34 | SMALL RUMINANTS | Female 1 г. 11 м. |
| 35 | SMALL RUMINANTS | Female 7 г. 1 м. |
| 36 | SMALL RUMINANTS | Female 1 г. 11 м. |
| 37 | SMALL RUMINANTS | Male 3 г. 1 м. |
| 38 | SMALL RUMINANTS | Female 2 г. 0 м. |
| 39 | SMALL RUMINANTS | Female 4 г. 2 м. |
| 40 | SMALL RUMINANTS | Female 7 г. 0 м. |
| 41 | SMALL RUMINANTS | Female 7 г. 0 м. |
| 42 | SMALL RUMINANTS | Female 1 г. 11 м. |
| 43 | SMALL RUMINANTS | Female 2 г. 4 м. |
| 44 | SMALL RUMINANTS | Female 5 г. 3 м. |
| 45 | SMALL RUMINANTS | Female 1 г. 11 м. |
| 46 | SMALL RUMINANTS | Female 2 г. 4 м. |
| 47 | SMALL RUMINANTS | Female 4 г. 5 м. |
| 48 | SMALL RUMINANTS | Female 1 г. 11 м. |
| 49 | SMALL RUMINANTS | Female 2 г. 0 м. |
| 50 | SMALL RUMINANTS | Female 5 г. 3 м. |

Sampling site: Zhezkazgan

GPS: Широта: 47.611291 Долгота: 67.852669

| **№**  **п/п** | Animal species | Sex and age |
| --- | --- | --- |
| 51 | CATTLE | Female 8 г. 4 м. |
| 52 | CATTLE | Female 1 г. 2 м. |
| 53 | CATTLE | Female 6 г. 1 м. |
| 54 | CATTLE | Female 9 г. 2 м. |
| 55 | CATTLE | Female 1 г. 2 м. |
| 56 | CATTLE | Female 6 г. 1 м. |
| 57 | CATTLE | Female 6 г. 3 м. |
| 58 | CATTLE | Female 1 г. 2 м. |
| 59 | CATTLE | Female 1 г. 2 м. |
| 60 | CATTLE | Female 8 г. 4 м. |
| 61 | CATTLE | Female 5 г. 6 м. |
| 62 | CATTLE | Male 2 г. 3 м. |
| 63 | CATTLE | Female 2 г. 4 м. |
| 64 | CATTLE | Female 6 г. 3 м. |
| 65 | CATTLE | Female 5 г. 3 м. |
| 66 | CATTLE | Female 2 г. 4 м. |
| 67 | CATTLE | Female 8 г. 4 м. |
| 68 | CATTLE | Female 7 г. 2 м. |
| 69 | CATTLE | Female 7 г. 2 м. |
| 70 | CATTLE | Female 8 г. 4 м. |
| 71 | CATTLE | Female 5 г. 3 м. |
| 72 | CATTLE | Female 6 г. 3 м. |
| 73 | CATTLE | Female 5 г. 3 м. |
| 74 | CATTLE | Female 8 г. 3 м. |
| 75 | CATTLE | Male 2 г. 3 м. |
| 76 | CATTLE | Female 5 г. 3 м. |
| 77 | CATTLE | Female 1 г 2 м. |
| 78 | CATTLE | Female 1 г. 2 м. |
| 79 | CATTLE | Male 4 г. 2 м. |
| 80 | CATTLE | Female 5 г. 3 м. |
| 81 | CATTLE | Male 6 г. 2 м. |
| 82 | CATTLE | Male 4 г. 2 м. |
| 83 | CATTLE | Female 5 г. 3 м. |
| 84 | CATTLE | Male 4 г. 2 м. |
| 85 | CATTLE | Female 5 г. 3 м. |
| 86 | CATTLE | Female 6 г. 3 м. |
| 87 | CATTLE | Female 6 г. 1 м. |
| 88 | CATTLE | Female 5 г. 5 м. |
| 89 | CATTLE | Female 6 г. 3 м. |
| 90 | CATTLE | Female 5 г. 3 м. |
| 91 | CATTLE | Female 6 г. 3 м. |
| 92 | CATTLE | Female 5 г. 3 м. |
| 93 | CATTLE | Male 4 г. 2 м. |
| 94 | CATTLE | Female 5 г. 3 м. |
| 95 | CATTLE | Female 5 г. 3 м. |
| 96 | CATTLE | Female 2 г. 4 м. |
| 97 | CATTLE | Female 5 г. 3 м. |
| 98 | CATTLE | Female 6 г. 1 м. |
| 99 | CATTLE | Female 5 г. 3 м. |
| 100 | CATTLE | Female 1 г. 2 м. |

Sampling site: Satbayev

GPS: Широта: 48.029723 Долгота: 67.634546

| **№**  **п/п** | Animal species | Sex and age |
| --- | --- | --- |
| 101 | SMALL RUMINANTS | Female 3 г. 8 м. |
| 102 | SMALL RUMINANTS | Female 1 г. 7 м. |
| 103 | SMALL RUMINANTS | Female 4 г. 6 м |
| 104 | SMALL RUMINANTS | Female 4 г. 6 м. |
| 105 | SMALL RUMINANTS | Female 4 г. 5 м. |
| 106 | SMALL RUMINANTS | Female 4 г. 6 м. |
| 107 | SMALL RUMINANTS | Female 5 г. 7 м. |
| 108 | SMALL RUMINANTS | Female 4 г. 5 м. |
| 109 | SMALL RUMINANTS | Female 3 г. 7 м. |
| 110 | SMALL RUMINANTS | Female 5 г. 7 м. |
| 111 | SMALL RUMINANTS | Female 3 г. 8 м. |
| 112 | SMALL RUMINANTS | Female 4 г. 7 м. |
| 113 | SMALL RUMINANTS | Female 5 г. 7 м. |
| 114 | SMALL RUMINANTS | Female 4 г. 6 м. |
| 115 | SMALL RUMINANTS | Female 4 г. 7 м. |
| 116 | SMALL RUMINANTS | Female 5 г. 7 м. |
| 117 | SMALL RUMINANTS | Female 4 г. 5 м. |
| 118 | SMALL RUMINANTS | Female 3 г. 7 м. |
| 119 | SMALL RUMINANTS | Female 5 г. 7 м. |
| 120 | SMALL RUMINANTS | Female 5 г. 7 м. |
| 121 | SMALL RUMINANTS | Female 5 г. 7 м. |
| 122 | SMALL RUMINANTS | Female 5 г. 7 м. |
| 123 | SMALL RUMINANTS | Female 4 г. 6 м. |
| 124 | SMALL RUMINANTS | Female 5 г. 7 м. |
| 125 | SMALL RUMINANTS | Female 4 г. 7 м. |
| 126 | SMALL RUMINANTS | Female 5 г. 7 м. |
| 127 | SMALL RUMINANTS | Female 4 г. 6 м. |
| 128 | SMALL RUMINANTS | Female 4 г. 7 м. |
| 129 | SMALL RUMINANTS | Female 5 г. 7 м. |
| 130 | SMALL RUMINANTS | Female 5 г. 7 м. |
| 131 | SMALL RUMINANTS | Female 4 г. 5 м. |
| 132 | SMALL RUMINANTS | Female 4 г. 7 м. |
| 133 | SMALL RUMINANTS | Female 3 г. 7 м. |
| 134 | SMALL RUMINANTS | Female 4 г. 5 м. |
| 135 | SMALL RUMINANTS | Female 5 г. 7 м. |
| 136 | SMALL RUMINANTS | Female 5 г. 7 м. |
| 137 | SMALL RUMINANTS | Female 4 г. 6 м. |
| 138 | SMALL RUMINANTS | Female 5 г. 7 м. |
| 139 | SMALL RUMINANTS | Female 5 г. 7 м. |
| 140 | SMALL RUMINANTS | Female 4 г. 5 м. |
| 141 | SMALL RUMINANTS | Female 4 г. 6 м. |
| 142 | SMALL RUMINANTS | Female 4 г. 5 м. |
| 143 | SMALL RUMINANTS | Female 5 г. 7 м. |
| 144 | SMALL RUMINANTS | Female 4 г. 5 м. |
| 145 | SMALL RUMINANTS | Female 5 г. 7 м. |
| 146 | SMALL RUMINANTS | Female 3 г. 7 м. |
| 147 | SMALL RUMINANTS | Female 4 г. 6 м. |
| 148 | SMALL RUMINANTS | Female 3 г. 7 м. |
| 149 | SMALL RUMINANTS | Female 4 г. 6 м. |
| 150 | SMALL RUMINANTS | Female 4 г. 5 м. |

Sampling site: Satbayev

GPS: Широта: 48.029723 Долгота: 67.634546

| **№**  **п/п** | Animal species | Sex and age |
| --- | --- | --- |
| 151 | CATTLE | Female 1 г. 1 м. |
| 152 | CATTLE | Female 4 г. 1 м. |
| 153 | CATTLE | Female 6 г. 0 м. |
| 154 | CATTLE | Female 5 г. 11 м. |
| 155 | CATTLE | Female 10 г. 0 м. |
| 156 | CATTLE | Female 2 г. 3 м. |
| 157 | CATTLE | Female 3 г. 9 м. |
| 158 | CATTLE | Female 11 г. 3 м. |
| 159 | CATTLE | Female 1 г. 3 м. |
| 160 | CATTLE | Female 1г. 3 м. |
| 161 | CATTLE | Female 3 г. 2 м. |
| 162 | CATTLE | Male 3 г. 7 м. |
| 163 | CATTLE | Female 3 г. 0 м. |
| 164 | CATTLE | Female 3 г. 2 м. |
| 165 | CATTLE | Female 3 г. 8 м. |
| 166 | CATTLE | Female 3 г 3 м. |
| 167 | CATTLE | Female 1 г. 3 м. |
| 168 | CATTLE | Female 3 г. 8 м |
| 169 | CATTLE | Female 1 г. 3 м. |
| 170 | CATTLE | Female 1 г. 3 м. |
| 171 | CATTLE | Female 7 г. 2 м. |
| 172 | CATTLE | Female 2 г. 1 м. |
| 173 | CATTLE | Female 1 г. 8 м. |
| 174 | CATTLE | Female 2 г. 1 м. |
| 175 | CATTLE | Male 1 г. 8 м. |
| 176 | CATTLE | Female 8 г. 11 м. |
| 177 | CATTLE | Female 8 г. 0 м. |
| 178 | CATTLE | Female 2 г 11 м. |
| 179 | CATTLE | Female 8 г. 11 м. |
| 180 | CATTLE | Female 2 г. 0 м. |
| 181 | CATTLE | Male 3 г. 10 м. |
| 182 | CATTLE | Female 2 г. 10 м. |
| 183 | CATTLE | Female 3 г. 11 м. |
| 184 | CATTLE | Female 1 г. 3 м. |
| 185 | CATTLE | Female 2 г. 0 м. |
| 186 | CATTLE | Female 2 г. 0 м. |
| 187 | CATTLE | Female 2 г. 1 м. |
| 188 | CATTLE | Female 2 г. 0 м. |
| 189 | CATTLE | Female 3 г. 0 м. |
| 190 | CATTLE | Male 3 г. 10 м. |
| 191 | CATTLE | Male 7 г. 11 м. |
| 192 | CATTLE | Male 1 г. 3 м. |
| 193 | CATTLE | Female 1 г. 1 м. |
| 194 | CATTLE | Female 2 г. 2 м. |
| 195 | CATTLE | Female 1 г. 1 м. |
| 196 | CATTLE | Female 1 г. 1 м. |
| 197 | CATTLE | Male 2 г. 0 м. |
| 198 | CATTLE | Female 1 г. 1 м. |
| 199 | CATTLE | Female 7 г. 1 м. |
| 200 | CATTLE | Female 10 г. 1 м. |

Sampling site: Ulytau

GPS: Широта: 48.830480 Долгота: 68.412196

| **№**  **п/п** | Animal species | Sex and age |
| --- | --- | --- |
| 201 | SMALL RUMINANTS | Female 4 г. 1 м. |
| 202 | SMALL RUMINANTS | Female 1 г. 1 м. |
| 203 | SMALL RUMINANTS | Female 4 г. 1 м. |
| 204 | SMALL RUMINANTS | Female 4 г. 1 м. |
| 205 | SMALL RUMINANTS | Female 1 г. 9 м. |
| 206 | SMALL RUMINANTS | Female 4 г. 1 м. |
| 207 | SMALL RUMINANTS | Female 1 г. 1 м. |
| 208 | SMALL RUMINANTS | Female 4 г. 10 м. |
| 209 | SMALL RUMINANTS | Female 1 г. 9 м. |
| 210 | SMALL RUMINANTS | Female 1 г. 9 м. |
| 211 | SMALL RUMINANTS | Female 4 г. 1 м. |
| 212 | SMALL RUMINANTS | Female 4 г. 1 м. |
| 213 | SMALL RUMINANTS | Female 4 г. 1 м. |
| 214 | SMALL RUMINANTS | Female 4 г. 1 м. |
| 215 | SMALL RUMINANTS | Female 4 г. 10 м. |
| 216 | SMALL RUMINANTS | Female 4 г. 1 м. |
| 217 | SMALL RUMINANTS | Female 4 г. 10 м. |
| 218 | SMALL RUMINANTS | Female 4 г. 10 м. |
| 219 | SMALL RUMINANTS | Female 1 г. 9 м. |
| 220 | SMALL RUMINANTS | Female 4 г. 1 м. |
| 221 | SMALL RUMINANTS | Female 4 г. 1 м. |
| 222 | SMALL RUMINANTS | Female 1 г. 1 м. |
| 223 | SMALL RUMINANTS | Female 1 г. 9 м. |
| 224 | SMALL RUMINANTS | Female 4 г. 10 м. |
| 225 | SMALL RUMINANTS | Female 1 г. 9 м. |
| 226 | SMALL RUMINANTS | Female 1 г. 9 м. |
| 227 | SMALL RUMINANTS | Female 1 г. 9 м. |
| 228 | SMALL RUMINANTS | Female 4 г. 1 м. |
| 229 | SMALL RUMINANTS | Female 4 г. 10 м. |
| 230 | SMALL RUMINANTS | Female 4 г. 10 м. |
| 231 | SMALL RUMINANTS | Female 4 г. 10 м. |
| 232 | SMALL RUMINANTS | Female 1 г. 9 м. |
| 233 | SMALL RUMINANTS | Female 4 г. 1 м. |
| 234 | SMALL RUMINANTS | Female 4 г. 1 м. |
| 235 | SMALL RUMINANTS | Female 1 г.1 м. |
| 236 | SMALL RUMINANTS | Female 4 г. 10 м. |
| 237 | SMALL RUMINANTS | Female 4 г. 1 м. |
| 238 | SMALL RUMINANTS | Female 4 г. 1 м. |
| 239 | SMALL RUMINANTS | Female 1 г. 9 м. |
| 240 | SMALL RUMINANTS | Female 4 г. 1 м. |
| 241 | SMALL RUMINANTS | Female 1 г. 1 м. |
| 242 | SMALL RUMINANTS | Female 4 г. 10 м. |
| 243 | SMALL RUMINANTS | Female 1 г. 9 м. |
| 244 | SMALL RUMINANTS | Female 1 г. 9 м. |
| 245 | SMALL RUMINANTS | Female 4 г. 10 м. |
| 246 | SMALL RUMINANTS | Female 4 г. 10 м. |
| 247 | SMALL RUMINANTS | Female 4 г. 1 м. |
| 248 | SMALL RUMINANTS | Female 1 г. 1 м. |
| 249 | SMALL RUMINANTS | Female 4 г. 10 м. |
| 250 | SMALL RUMINANTS | Female 4 г. 10 м. |

Sampling site: Ulytau

GPS: Широта: 48.830480 Долгота: 68.412196

| **№**  **п/п** | Animal species | Sex and age |
| --- | --- | --- |
| 251 | CATTLE | Female 4 г. 9 м. |
| 252 | CATTLE | Female 3 г. 2 м. |
| 253 | CATTLE | Female 3 г. 2 м. |
| 254 | CATTLE | Female2 г. 1 м. |
| 255 | CATTLE | Female 3 г. 2 м. |
| 256 | CATTLE | Female 9 г. 1 м. |
| 257 | CATTLE | Female 4 г. 9 м. |
| 258 | CATTLE | Female 3 г. 2 м. |
| 259 | CATTLE | Female 4 г. 9 м. |
| 260 | CATTLE | Female 9 г. 1 м. |
| 261 | CATTLE | Female 3 г. 2 м. |
| 262 | CATTLE | Female 9 г. 1 м. |
| 263 | CATTLE | Female 3 г. 2 м. |
| 264 | CATTLE | Female 1 г. 3 м. |
| 265 | CATTLE | Female 4 г. 2 м. |
| 266 | CATTLE | Female 8 г. 1 м. |
| 267 | CATTLE | Female 1 г. 3 м. |
| 268 | CATTLE | Female 8 г. 2 м. |
| 269 | CATTLE | Female 1 г. 3 м. |
| 270 | CATTLE | Female 3 г. 2 м. |
| 271 | CATTLE | Female 2 г. 1 м. |
| 272 | CATTLE | Female 9 г. 1 м. |
| 273 | CATTLE | Female 1 г. 3 м. |
| 274 | CATTLE | Female 2 г. 1 м. |
| 275 | CATTLE | Female 2 г. 1 м. |
| 276 | CATTLE | Female 9 г. 1 м. |
| 277 | CATTLE | Female 1 г. 3 м. |
| 278 | CATTLE | Female3 г. 2 м. |
| 279 | CATTLE | Female 1 г. 3 м. |
| 280 | CATTLE | Female 9 г. 1 м. |
| 281 | CATTLE | Female 8 г. 1 м. |
| 282 | CATTLE | Female 3 г. 2 м. |
| 283 | CATTLE | Female 2 г. 1 м. |
| 284 | CATTLE | Female 1 г. 1 м. |
| 285 | CATTLE | Female 1 г. 3 м. |
| 286 | CATTLE | Female 1 г. 3 м. |
| 287 | CATTLE | Female 1 г. 3 м. |
| 288 | CATTLE | Female 1 г. 3 м. |
| 189 | CATTLE | Female 1 г. 3 м. |
| 290 | CATTLE | Female 1 г. 3 м. |
| 291 | CATTLE | Female 9 г. 2 м. |
| 292 | CATTLE | Female 8 г. 1 м. |
| 293 | CATTLE | Female 8 г. 2 м. |
| 294 | CATTLE | Female 9 г. 1 м. |
| 295 | CATTLE | Female 2 г. 1 м. |
| 296 | CATTLE | Female 1 г. 3 м. |
| 297 | CATTLE | Female 4 г. 2 м. |
| 298 | CATTLE | Female 1 г. 3 м. |
| 299 | CATTLE | Female 3 г. 2 м. |
| 300 | CATTLE | Female 4 г. 9 м. |

Sampling site: Zhanaarka

GPS: Широта: 48.756678 Долгота: 71.670293

| **№**  **п/п** | Animal species | Sex and age |
| --- | --- | --- |
| 301 | SMALL RUMINANTS | Female, 3 г. 2 м. |
| 302 | SMALL RUMINANTS | Female, 3 г. 2 м. |
| 303 | SMALL RUMINANTS | Female, 3 г. 2 м. |
| 304 | SMALL RUMINANTS | Female, 3 г. 2 м. |
| 305 | SMALL RUMINANTS | Female, 3 г. 2 м. |
| 306 | SMALL RUMINANTS | Female, 3 г. 2 м. |
| 307 | SMALL RUMINANTS | Female, 3 г. 2 м. |
| 308 | SMALL RUMINANTS | Female, 3 г. 2 м. |
| 309 | SMALL RUMINANTS | Female, 3 г. 2 м. |
| 310 | SMALL RUMINANTS | Female, 3 г. 2 м. |
| 311 | SMALL RUMINANTS | Female, 3 г. 2 м. |
| 312 | SMALL RUMINANTS | Female, 3 г. 2 м. |
| 213 | SMALL RUMINANTS | Female, 3 г. 2 м. |
| 314 | SMALL RUMINANTS | Female, 3 г. 2 м. |
| 315 | SMALL RUMINANTS | Female, 3 г. 2 м. |
| 316 | SMALL RUMINANTS | Female, 3 г. 2 м. |
| 317 | SMALL RUMINANTS | Female, 3 г. 2 м. |
| 318 | SMALL RUMINANTS | Female, 3 г. 2 м. |
| 319 | SMALL RUMINANTS | Female, 3 г. 2 м. |
| 320 | SMALL RUMINANTS | Female, 3 г. 2 м. |
| 321 | SMALL RUMINANTS | Female, 3 г. 2 м. |
| 322 | SMALL RUMINANTS | Female, 3 г. 2 м. |
| 323 | SMALL RUMINANTS | Female, 3 г. 2 м. |
| 324 | SMALL RUMINANTS | Female, 3 г. 2 м. |
| 325 | SMALL RUMINANTS | Female, 3 г. 2 м. |
| 326 | SMALL RUMINANTS | Female, 3 г. 2 м. |
| 327 | SMALL RUMINANTS | Female, 3 г. 2 м. |
| 328 | SMALL RUMINANTS | Female, 3 г. 2 м. |
| 329 | SMALL RUMINANTS | Female, 3 г. 2 м. |
| 330 | SMALL RUMINANTS | Female, 3 г. 2 м. |
| 331 | SMALL RUMINANTS | Female, 3 г. 2 м. |
| 332 | SMALL RUMINANTS | Female, 3 г. 2 м. |
| 333 | SMALL RUMINANTS | Female, 3 г. 2 м. |
| 334 | SMALL RUMINANTS | Female, 3 г. 2 м. |
| 335 | SMALL RUMINANTS | Female, 3 г. 1 м. |
| 336 | SMALL RUMINANTS | Female, 3 г. 2 м. |
| 337 | SMALL RUMINANTS | Female, 3 г. 2 м. |
| 338 | SMALL RUMINANTS | Female, 3 г. 2 м. |
| 339 | SMALL RUMINANTS | Female, 3 г. 1 м. |
| 340 | SMALL RUMINANTS | Female, 3 г. 2 м. |
| 341 | SMALL RUMINANTS | Female, 3 г. 2 м. |
| 342 | SMALL RUMINANTS | Female, 3 г. 2 м. |
| 343 | SMALL RUMINANTS | Female, 3 г. 2 м. |
| 344 | SMALL RUMINANTS | Female, 3 г. 2 м. |
| 345 | SMALL RUMINANTS | Female, 3 г. 2 м. |
| 346 | SMALL RUMINANTS | Female, 3 г. 2 м. |
| 347 | SMALL RUMINANTS | Female, 3 г. 2 м. |
| 348 | SMALL RUMINANTS | Female, 3 г. 2 м. |
| 349 | SMALL RUMINANTS | Female, 3 г. 2 м. |
| 350 | SMALL RUMINANTS | Female, 3 г. 2 м. |

Sampling site: Zhanaarka

GPS: Широта: 48.756678 Долгота: 71.670293

| **№**  **п/п** | Animal species | Sex and age |
| --- | --- | --- |
|  | CATTLE | Female, 2 г. 1 м. |
| 352 | CATTLE | Female, 2 г. 1 м. |
| 353 | CATTLE | Female, 2 г. 1 м. |
| 354 | CATTLE | Female, 2 г. 1 м. |
| 355 | CATTLE | Female, 2 г. 1 м. |
| 356 | CATTLE | Female, 2 г. 3 м. |
| 357 | CATTLE | Female, 2 г. 3 м. |
| 358 | CATTLE | Female, 2 г. 3 м. |
| 359 | CATTLE | Female, 2 г. 3 м. |
| 360 | CATTLE | Female, 2 г. 4 м. |
| 361 | CATTLE | Male, 2 г. 3 м. |
| 362 | CATTLE | Male, 2 г. 3 м. |
| 363 | CATTLE | Male, 2 г. 3 м. |
| 364 | CATTLE | Male, 2 г. 2 м. |
| 365 | CATTLE | Male, 2 г. 3 м. |
| 366 | CATTLE | Female, 2 г. 3 м. |
| 367 | CATTLE | Male, 2 г. 3 м. |
| 368 | CATTLE | Male, 2 г. 3 м. |
| 369 | CATTLE | Male, 2 г. 3 м. |
| 370 | CATTLE | Female, 2 г. 3 м. |
| 371 | CATTLE | Female, 3 г. 2 м. |
| 372 | CATTLE | Female, 3 г. 2 м. |
| 373 | CATTLE | Female, 6 г. 0 м. |
| 374 | CATTLE | Female, 6 г. 0 м. |
| 375 | CATTLE | Female, 6 г. 0 м. |
| 376 | CATTLE | Female, 3 г. 4 м. |
| 377 | CATTLE | Male, 3 г. 4 м. |
| 378 | CATTLE | Female, 4 г. 0 м. |
| 379 | CATTLE | Female, 4 г. 0 м. |
| 380 | CATTLE | Female, 4 г.1 м. |
| 381 | CATTLE | Female, 4 г. 1 м. |
| 382 | CATTLE | Female, 4 г. 1 м. |
| 383 | CATTLE | Female, 4 г. 1 м. |
| 384 | CATTLE | Female, 4 г. 1 м |
| 385 | CATTLE | Female, 4 г. 1 м. |
| 386 | CATTLE | Female, 2 г. 1 м. |
| 387 | CATTLE | Female, 2 г. 1 м. |
| 388 | CATTLE | Female, 2 г. 1 м. |
| 389 | CATTLE | Female, 2 г. 1 м. |
| 390 | CATTLE | Female, 2 г. 1 м. |
| 391 | CATTLE | Female, 2 г. 1 м. |
| 392 | CATTLE | Male, 2 г. 1 м. |
| 393 | CATTLE | Male, 2 г. 1 м. |
| 394 | CATTLE | Male, 2 г. 1 м. |
| 395 | CATTLE | Male, 2 г. 1 м. |
| 396 | CATTLE | Male, 2 г. 1 м. |
| 397 | CATTLE | Male, 2 г. 1 м. |
| 398 | CATTLE | Male, 2 г. 1 м. |
| 399 | CATTLE | Male, 2 г. 1 м. |
| 400 | CATTLE | Male, 2 г. 1 м. |

Sampling site: Karazhal

GPS: Широта: 48.009291 Долгота: 70.774834

| **№**  **п/п** | Animal species | Sex and age |
| --- | --- | --- |
| 401 | SMALL RUMINANTS | Female, 4 г. 3 м. |
| 402 | SMALL RUMINANTS | Female, 4 г. 3 м. |
| 403 | SMALL RUMINANTS | Male, 4 г. 3 м. |
| 404 | SMALL RUMINANTS | Female, 1 г. 4 м. |
| 405 | SMALL RUMINANTS | Male, 1 г. 4 м. |
| 406 | SMALL RUMINANTS | Male, 1 г. 4 м. |
| 407 | SMALL RUMINANTS | Female, 1 г. 4 м. |
| 408 | SMALL RUMINANTS | Female, 1 г. 4 м. |
| 409 | SMALL RUMINANTS | Female, 1 г. 4 м. |
| 410 | SMALL RUMINANTS | Male, 7 г. 1 м. |
| 411 | SMALL RUMINANTS | Male, 7 г. 1 м. |
| 412 | SMALL RUMINANTS | Female, 8 г. 2 м. |
| 413 | SMALL RUMINANTS | Male, 8 г. 3 м. |
| 414 | SMALL RUMINANTS | Male, 8 г. 3 м. |
| 415 | SMALL RUMINANTS | Male, 8 г. 2 м. |
| 416 | SMALL RUMINANTS | Male, 9 г. 1 м. |
| 417 | SMALL RUMINANTS | Female, 7 г. 9 м. |
| 418 | SMALL RUMINANTS | Female, 7 г. 9 м. |
| 419 | SMALL RUMINANTS | Female, 9 г. 1 м. |
| 420 | SMALL RUMINANTS | Male 7 г. 10 м. |
| 421 | SMALL RUMINANTS | Male 9 г. 0 м. |
| 422 | SMALL RUMINANTS | Male 7 г. 10 м. |
| 423 | SMALL RUMINANTS | Female 9 г. 2 м. |
| 424 | SMALL RUMINANTS | Male 7 г. 10 м. |
| 425 | SMALL RUMINANTS | Male 9 г. 2 м. |
| 426 | SMALL RUMINANTS | Female, 7 г. 9 м. |
| 427 | SMALL RUMINANTS | Female 10 г. 9 м. |
| 428 | SMALL RUMINANTS | Male 7 г. 10 м. |
| 429 | SMALL RUMINANTS | Female 10 г. 9 м. |
| 430 | SMALL RUMINANTS | Female 9 г. 3 м. |
| 431 | SMALL RUMINANTS | Female, 4 г. 2 м. |
| 432 | SMALL RUMINANTS | Female, 4 г. 2 м. |
| 433 | SMALL RUMINANTS | Female, 4 г. 2 м. |
| 434 | SMALL RUMINANTS | Female, 4 г. 2 м. |
| 435 | SMALL RUMINANTS | Male, 4 г. 2 м. |
| 436 | SMALL RUMINANTS | Female, 4 г. 2 м. |
| 437 | SMALL RUMINANTS | Female, 4 г. 2 м. |
| 438 | SMALL RUMINANTS | Female, 4 г. 2 м. |
| 439 | SMALL RUMINANTS | Female, 4 г. 2 м. |
| 440 | SMALL RUMINANTS | Female, 4 г. 2 м. |
| 441 | SMALL RUMINANTS | Female, 4 г. 2 м. |
| 442 | SMALL RUMINANTS | Female, 4 г. 2 м. |
| 443 | SMALL RUMINANTS | Female, 4 г. 2 м. |
| 444 | SMALL RUMINANTS | Female, 4 г. 2 м. |
| 445 | SMALL RUMINANTS | Female, 4 г. 2 м. |
| 446 | SMALL RUMINANTS | Female, 8 г. 1 м. |
| 447 | SMALL RUMINANTS | Female, 8 г. 1 м. |
| 448 | SMALL RUMINANTS | Female, 7 г 2 м. |
| 449 | SMALL RUMINANTS | Female, 7 г. 2 м. |
| 450 | SMALL RUMINANTS | Female, 8 г. 1 м. |

Sampling site: Karazhal

GPS: Широта: 48.009291 Долгота: 70.774834

| **№**  **п/п** | Animal species | Sex and age |
| --- | --- | --- |
| 451 | CATTLE | Male, 1 г. 1 м. |
| 452 | CATTLE | Male, 1 г. 1 м. |
| 453 | CATTLE | Female, 1 г. 1 м. |
| 454 | CATTLE | Female 1 г. 11 м. |
| 455 | CATTLE | Female 1 г. 11 м. |
| 456 | CATTLE | Male 1 г. 11 м. |
| 457 | CATTLE | Female 1 г. 0 м. |
| 458 | CATTLE | Male 1 г. 0 м. |
| 459 | CATTLE | Female 1 г. 11 м. |
| 460 | CATTLE | Female 1 г. 11 м. |
| 461 | CATTLE | Female, 1 г. 6 м. |
| 462 | CATTLE | Male, 1 г. 6 м. |
| 463 | CATTLE | Female, 1 г. 6 м. |
| 464 | CATTLE | Female, 1 г. 6 м. |
| 465 | CATTLE | Female, 1 г. 6 м. |
| 466 | CATTLE | Female, 1 г. 6 м. |
| 467 | CATTLE | Female, 1 г. 6 м. |
| 468 | CATTLE | Male, 1 г. 6 м. |
| 469 | CATTLE | Female, 1 г. 6 м. |
| 470 | CATTLE | Female, 1 г. 6 м. |
| 471 | CATTLE | Female, 1 г. 6 м. |
| 472 | CATTLE | Female, 1 г. 1 м.. |
| 473 | CATTLE | Male, 1 г. 1 м. |
| 474 | CATTLE | Female, 3 г. 1 м. |
| 475 | CATTLE | Male, 3 г. 1 м. |
| 476 | CATTLE | Female, 1 г. 2 м. |
| 477 | CATTLE | Female, 3 г. 7 м. |
| 478 | CATTLE | Female, 4 г. 1 м. |
| 479 | CATTLE | Female 4 г. 11 м. |
| 480 | CATTLE | Female 4 г. 11 м. |
| 481 | CATTLE | Female 4 г. 11 м. |
| 482 | CATTLE | Female, 1 г. 1 м. |
| 483 | CATTLE | Female 1 г. 11 м. |
| 484 | CATTLE | Female 3 г. 11 м. |
| 485 | CATTLE | Female, 1 г. 0 м. |
| 486 | CATTLE | Female, 1 г. 1 м. |
| 487 | CATTLE | Female, 2 г. 0 м. |
| 488 | CATTLE | Female, 3 г. 1 м. |
| 489 | CATTLE | Female, 1 г. 2 м. |
| 490 | CATTLE | Female, 1 г. 2 м. |
| 491 | CATTLE | Male 1 г. 11 м. |
| 492 | CATTLE | Male 1 г. 11 м. |
| 493 | CATTLE | Female 1 г. 11 м. |
| 494 | CATTLE | Male 1 г. 11 м. |
| 495 | CATTLE | Female 5 г. 11 м. |
| 496 | CATTLE | Female 7 г. 10 м. |
| 497 | CATTLE | Female 1 г. 2 м. |
| 498 | CATTLE | Female 3 г. 11 м. |
| 499 | CATTLE | Female 3 г. 11 м. |
| 500 | CATTLE | Female 3 г. 11 м. |

Sampling site: Shet

GPS: Широта: 48.849389 Долгота: 72.735582

| **№**  **п/п** | Animal species | Sex and age |
| --- | --- | --- |
| 501 | SMALL RUMINANTS | Female, 5 л. 0 м. |
| 502 | SMALL RUMINANTS | Male, 1 г. 3 м |
| 503 | SMALL RUMINANTS | Female, 4 г. 0 м. |
| 504 | SMALL RUMINANTS | Female, 3 г. 2 м. |
| 505 | SMALL RUMINANTS | Male, 1 г. 3 м. |
| 506 | SMALL RUMINANTS | Male, 1 г. 3 м. |
| 507 | SMALL RUMINANTS | Female, 4 г. 0 м. |
| 508 | SMALL RUMINANTS | Female, 4 г. 0 м. |
| 509 | SMALL RUMINANTS | Female, 4 г. 0 м. |
| 510 | SMALL RUMINANTS | Female, 4 г. 0 м. |
| 511 | SMALL RUMINANTS | Male, 1 г. 3 м. |
| 512 | SMALL RUMINANTS | Female, 4 г. 0 м. |
| 513 | SMALL RUMINANTS | Female, 4 г. 0 м. |
| 514 | SMALL RUMINANTS | Male, 1 г. 3 м. |
| 515 | SMALL RUMINANTS | Female, 4 г. 0 м. |
| 516 | SMALL RUMINANTS | Female, 4 г. 0 м. |
| 517 | SMALL RUMINANTS | Male 1 г. 3 м. |
| 518 | SMALL RUMINANTS | Male, 1 г. 3 м. |
| 519 | SMALL RUMINANTS | Female, 4 г. 0 м. |
| 520 | SMALL RUMINANTS | Male 1 г. 3 м. |
| 521 | SMALL RUMINANTS | Male 1 г. 3 м. |
| 522 | SMALL RUMINANTS | Male 1 г. 3 м. |
| 523 | SMALL RUMINANTS | Female, 4 г. 0 м. |
| 524 | SMALL RUMINANTS | Female, 4 г. 0 м. |
| 525 | SMALL RUMINANTS | Female, 4 г. 0 м. |
| 526 | SMALL RUMINANTS | Female, 3 г. 2 м. |
| 527 | SMALL RUMINANTS | Male 1 г. 3 м. |
| 528 | SMALL RUMINANTS | Male 1 г. 3 м. |
| 529 | SMALL RUMINANTS | Female, 3 г. 2 м. |
| 530 | SMALL RUMINANTS | Female, 4 г. 0 м. |
| 531 | SMALL RUMINANTS | Male 1 г. 3 м. |
| 532 | SMALL RUMINANTS | Female, 3 г. 2 м. |
| 533 | SMALL RUMINANTS | Male 1 г. 3 м. |
| 534 | SMALL RUMINANTS | Female, 4 г. 0 м. |
| 535 | SMALL RUMINANTS | Female, 4 г. 0 м. |
| 536 | SMALL RUMINANTS | Female, 3 г. 2 м. |
| 537 | SMALL RUMINANTS | Male 1 г. 3 м. |
| 538 | SMALL RUMINANTS | Female, 3 г. 2 м. |
| 539 | SMALL RUMINANTS | Male 1 г. 3 м. |
| 540 | SMALL RUMINANTS | Female, 3 г. 2 м. |
| 541 | SMALL RUMINANTS | Female, 4 г. 0 м. |
| 542 | SMALL RUMINANTS | Female, 4 г. 0 м. |
| 543 | SMALL RUMINANTS | Male 1 г. 3 м. |
| 544 | SMALL RUMINANTS | Male 1 г. 3 м. |
| 545 | SMALL RUMINANTS | Female, 5 л. 0 м. |
| 546 | SMALL RUMINANTS | Male 1 г. 3 м. |
| 547 | SMALL RUMINANTS | Female, 4 г. 0 м. |
| 548 | SMALL RUMINANTS | Male 1 г. 3 м. |
| 549 | SMALL RUMINANTS | Female, 3 г. 2 м. |
| 550 | SMALL RUMINANTS | Male 1 г. 3 м. |

Sampling site: Shet

GPS: Широта: 48.849389 Долгота: 72.735582

| **№**  **п/п** | Animal species | Sex and age |
| --- | --- | --- |
| 551 | CATTLE | Male, 1 г. 3 м. |
| 552 | CATTLE | Male, 2 г. 4 м. |
| 553 | CATTLE | Female, 6 л. 2 м. |
| 554 | CATTLE | Male, 1 г. 3 м. |
| 555 | CATTLE | Male, 1 г. 3 м. |
| 556 | CATTLE | Female, 2 г. 4 м. |
| 557 | CATTLE | Female, 6 л. 2 м. |
| 558 | CATTLE | Female, 6 л. 2 м. |
| 559 | CATTLE | Female, 3 г. 3 м. |
| 560 | CATTLE | Female, 6 л. 2 м. |
| 561 | CATTLE | Female, 3 г. 3 м. |
| 562 | CATTLE | Female, 3 г. 3 м. |
| 563 | CATTLE | Male, 1 г. 3 м. |
| 564 | CATTLE | Female, 4 г. 0 м. |
| 565 | CATTLE | Female, 3 г. 3 м. |
| 566 | CATTLE | Female, 4 г. 0 м. |
| 567 | CATTLE | Female, 4 г. 4 м. |
| 568 | CATTLE | Female, 3 г. 3 м. |
| 569 | CATTLE | Female, 2 г. 3 м |
| 570 | CATTLE | Female, 4 г. 0 м. |
| 571 | CATTLE | Female, 2 г. 3 м |
| 572 | CATTLE | Male, 1 г. 3 м. |
| 573 | CATTLE | Male, 1 г. 3 м. |
| 574 | CATTLE | Male, 1 г. 3 м. |
| 575 | CATTLE | Female, 3 г. 3 м. |
| 576 | CATTLE | Female, 4 г. 4 м. |
| 577 | CATTLE | Male, 1 г. 3 м. |
| 578 | CATTLE | Female, 4 г. 0 м. |
| 579 | CATTLE | Female, 4 г. 4 м. |
| 580 | CATTLE | Male, 1 г. 3 м. |
| 581 | CATTLE | Female, 4 г. 0 м. |
| 582 | CATTLE | Female, 3 г. 3 м. |
| 583 | CATTLE | Female, 4 г. 4 м. |
| 584 | CATTLE | Male, 1 г. 3 м. |
| 585 | CATTLE | Female, 3 г. 3 м. |
| 586 | CATTLE | Female, 4 г. 4 м. |
| 587 | CATTLE | Male, 2 г. 3 м |
| 588 | CATTLE | Male, 1 г. 3 м. |
| 589 | CATTLE | Male, 1 г. 3 м. |
| 590 | CATTLE | Female, 3 г. 2 м. |
| 591 | CATTLE | Male, 1 г. 3 м. |
| 592 | CATTLE | Female, 3 г. 3 м. |
| 593 | CATTLE | Male, 1 г. 3 м. |
| 594 | CATTLE | Female, 7 л. 1 м. |
| 595 | CATTLE | Female, 1 г. 3 м. |
| 596 | CATTLE | Female, 7 л. 3 м. |
| 597 | CATTLE | Female, 4 г. 0 м. |
| 598 | CATTLE | Female, 3 г. 2 м. |
| 599 | CATTLE | Female, 4 г. 0 м. |
| 600 | CATTLE | Female, 3 г. 2 м. |

Sampling site: Aktogay

GPS: Широта: 48.308160 Долгота: 74.978354

| **№**  **п/п** | Animal species | Sex and age |
| --- | --- | --- |
| 601 | SMALL RUMINANTS | Female 2 г. 3 м. |
| 602 | SMALL RUMINANTS | Female 2 г. 3 м. |
| 603 | SMALL RUMINANTS | Female 2 г. 3 м. |
| 604 | SMALL RUMINANTS | Female 2 г. 3 м. |
| 605 | SMALL RUMINANTS | Female 2 г. 3 м. |
| 606 | SMALL RUMINANTS | Female 2 г. 3 м. |
| 607 | SMALL RUMINANTS | Female 3 г. 4 м. |
| 608 | SMALL RUMINANTS | Female 3 г. 4 м. |
| 609 | SMALL RUMINANTS | Female 3 г. 4 м. |
| 610 | SMALL RUMINANTS | Female 3 г. 4 м. |
| 611 | SMALL RUMINANTS | Female 3 г. 4 м. |
| 612 | SMALL RUMINANTS | Female 3 г. 4 м. |
| 613 | SMALL RUMINANTS | Female 3 г. 4 м. |
| 614 | SMALL RUMINANTS | Female 3 г. 4 м. |
| 615 | SMALL RUMINANTS | Female 3 г. 4 м. |
| 616 | SMALL RUMINANTS | Female 3 г. 4 м. |
| 617 | SMALL RUMINANTS | Female 3 г. 4 м. |
| 618 | SMALL RUMINANTS | Female 3 г. 4 м. |
| 619 | SMALL RUMINANTS | Female 3 г. 4 м. |
| 620 | SMALL RUMINANTS | Female 3 г. 4 м. |
| 621 | SMALL RUMINANTS | Male 0 г. 11 м. |
| 622 | SMALL RUMINANTS | Male 0 г. 11 м. |
| 623 | SMALL RUMINANTS | Female 4 г. 3 м. |
| 624 | SMALL RUMINANTS | Female 4 г. 3 м. |
| 625 | SMALL RUMINANTS | Female 4 г. 3 м. |
| 626 | SMALL RUMINANTS | Female 4 г. 3 м. |
| 627 | SMALL RUMINANTS | Female 4 г. 3 м. |
| 628 | SMALL RUMINANTS | Female 5 г. 3 м. |
| 629 | SMALL RUMINANTS | Female 6 г. 2 м. |
| 630 | SMALL RUMINANTS | Female 5 г. 3 м. |
| 631 | SMALL RUMINANTS | Female 5 г. 4 м. |
| 632 | SMALL RUMINANTS | Female 5 г. 4 м. |
| 633 | SMALL RUMINANTS | Female 5 г. 5 м. |
| 634 | SMALL RUMINANTS | Female 5 г. 3 м. |
| 635 | SMALL RUMINANTS | Female 5 г. 4 м. |
| 636 | SMALL RUMINANTS | Female 5 г. 4 м. |
| 637 | SMALL RUMINANTS | Female 5 г. 5 м. |
| 638 | SMALL RUMINANTS | Male 0 г. 11 м. |
| 639 | SMALL RUMINANTS | Male 0 г. 11 м. |
| 640 | SMALL RUMINANTS | Male 0 г. 11 м. |
| 641 | SMALL RUMINANTS | Female 5 г. 4 м. |
| 642 | SMALL RUMINANTS | Female 5 г. 4 м. |
| 643 | SMALL RUMINANTS | Female 6 г. 4 м. |
| 644 | SMALL RUMINANTS | Female 6 г. 4 м. |
| 645 | SMALL RUMINANTS | Female 6 г. 4 м. |
| 646 | SMALL RUMINANTS | Female 6 г. 4 м. |
| 647 | SMALL RUMINANTS | Female 6 г. 4 м. |
| 648 | SMALL RUMINANTS | Female 6 г. 4 м. |
| 649 | SMALL RUMINANTS | Female 6 г. 4 м. |
| 650 | SMALL RUMINANTS | Female 6 г. 4 м. |

Sampling site: Aktogay

GPS: Широта: 48.308160 Долгота: 74.978354

| **№**  **п/п** | Animal species | Sex and age |
| --- | --- | --- |
| 651 | CATTLE | Female 2 г. 2 м. |
| 652 | CATTLE | Female 2 г. 2 м. |
| 653 | CATTLE | Female 3 г. 3 м. |
| 654 | CATTLE | Female 3 г. 3 м. |
| 655 | CATTLE | Female 3 г. 3 м. |
| 656 | CATTLE | Female 8 г. 4 м. |
| 657 | CATTLE | Female 6 г. 5 м. |
| 658 | CATTLE | Female 2 г. 2 м. |
| 659 | CATTLE | Female 2 г. 2 м. |
| 660 | CATTLE | Female 5 г. 0 м. |
| 661 | CATTLE | Male 1 г. 0 м. |
| 662 | CATTLE | Male 1 г. 0 м. |
| 663 | CATTLE | Male 1 г. 0 м. |
| 664 | CATTLE | Male 1 г. 0 м. |
| 665 | CATTLE | Male 1 г. 0 м. |
| 666 | CATTLE | Male 1 г. 0 м. |
| 667 | CATTLE | Female 3 г. 4 м. |
| 668 | CATTLE | Female 3 г. 4 м. |
| 669 | CATTLE | Female 3 г. 4 м. |
| 670 | CATTLE | Female 3 г. 4 м. |
| 671 | CATTLE | Female 3 г. 4 м. |
| 672 | CATTLE | Female 4 г. 4 м. |
| 673 | CATTLE | Female 6 г. 3 м. |
| 674 | CATTLE | Female 7 г. 2 м. |
| 675 | CATTLE | Female 7 г. 2 м. |
| 676 | CATTLE | Male 1 г. 0 м. |
| 677 | CATTLE | Male 1 г. 0 м. |
| 678 | CATTLE | Male 1 г. 0 м. |
| 679 | CATTLE | Male 1 г. 0 м. |
| 680 | CATTLE | Male 1 г. 0 м. |
| 681 | CATTLE | Male 1 г. 9 м. |
| 682 | CATTLE | Female 2 г. 4 м. |
| 683 | CATTLE | Female 3 г. 11 м. |
| 684 | CATTLE | Female 3 г. 11 м. |
| 685 | CATTLE | Female 3 г. 11 м. |
| 686 | CATTLE | Female 3 г. 11 м. |
| 687 | CATTLE | Female 3 г. 11 м. |
| 688 | CATTLE | Female 3 г. 11 м. |
| 689 | CATTLE | Female 3 г. 11 м. |
| 690 | CATTLE | Female 4 г. 2 м. |
| 691 | CATTLE | Male 1 г. 0 м. |
| 692 | CATTLE | Male 1 г. 9 м. |
| 693 | CATTLE | Male 1 г. 9 м. |
| 694 | CATTLE | Female 2 г. 4 м. |
| 695 | CATTLE | Female 2 г. 4 м. |
| 696 | CATTLE | Female 2 г. 4 м. |
| 697 | CATTLE | Female 3 г. 2 м. |
| 698 | CATTLE | Female 3 г. 2 м. |
| 699 | CATTLE | Female 3 г. 2 м. |
| 700 | CATTLE | Female 3 г. 11 м. |
