# Supplementary material for: Integrated surveillance, virus isolation and phylogenetic characterization of Crimean-Congo hemorrhagic fever virus in Central Kazakhstan
Source: Front Vet Sci. 2026 Jul 16;13:1879322. doi: 10.3389/fvets.2026.1879322 (PMC13421902; doi:10.3389/fvets.2026.1879322)
Supplement: Supplementary file 2 [file Supplementary_file_1.docx]

| № | Цвет | **Pool ID** | Тип | CT |
| --- | --- | --- | --- | --- |
| 1 | 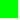 | Pool 1 | Sample |  |
| 2 | 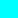 | Pool 2 | Sample |  |
| 3 | 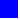 | Pool 3 | Sample |  |
| 4 | 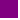 | Pool 4 | Sample |  |
| 5 | 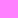 | Pool 5 | Sample |  |
| 6 | 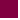 | Pool 6 | Sample |  |
| 7 | 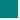 | Pool 7 | Sample |  |
| 8 | 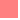 | Pool 8 | Sample | 30,77 |
| 9 | 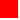 | PC | Positive control | 25,73 |
| 10 | 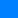 | NC | Negative control |  |


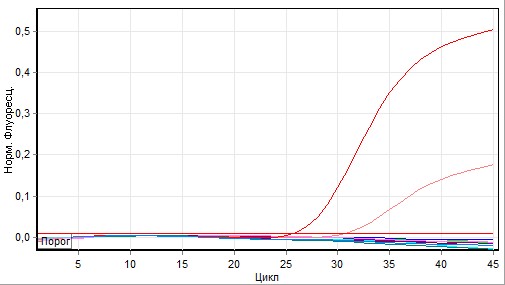


Results of RT-PCR CCHFV RNA
