# Supplementary material for: Integrated surveillance, virus isolation and phylogenetic characterization of Crimean-Congo hemorrhagic fever virus in Central Kazakhstan
Source: Front Vet Sci. 2026 Jul 16;13:1879322. doi: 10.3389/fvets.2026.1879322 (PMC13421902; doi:10.3389/fvets.2026.1879322)
Supplement: Supplementary file 3 [file Supplementary_file_2.docx]

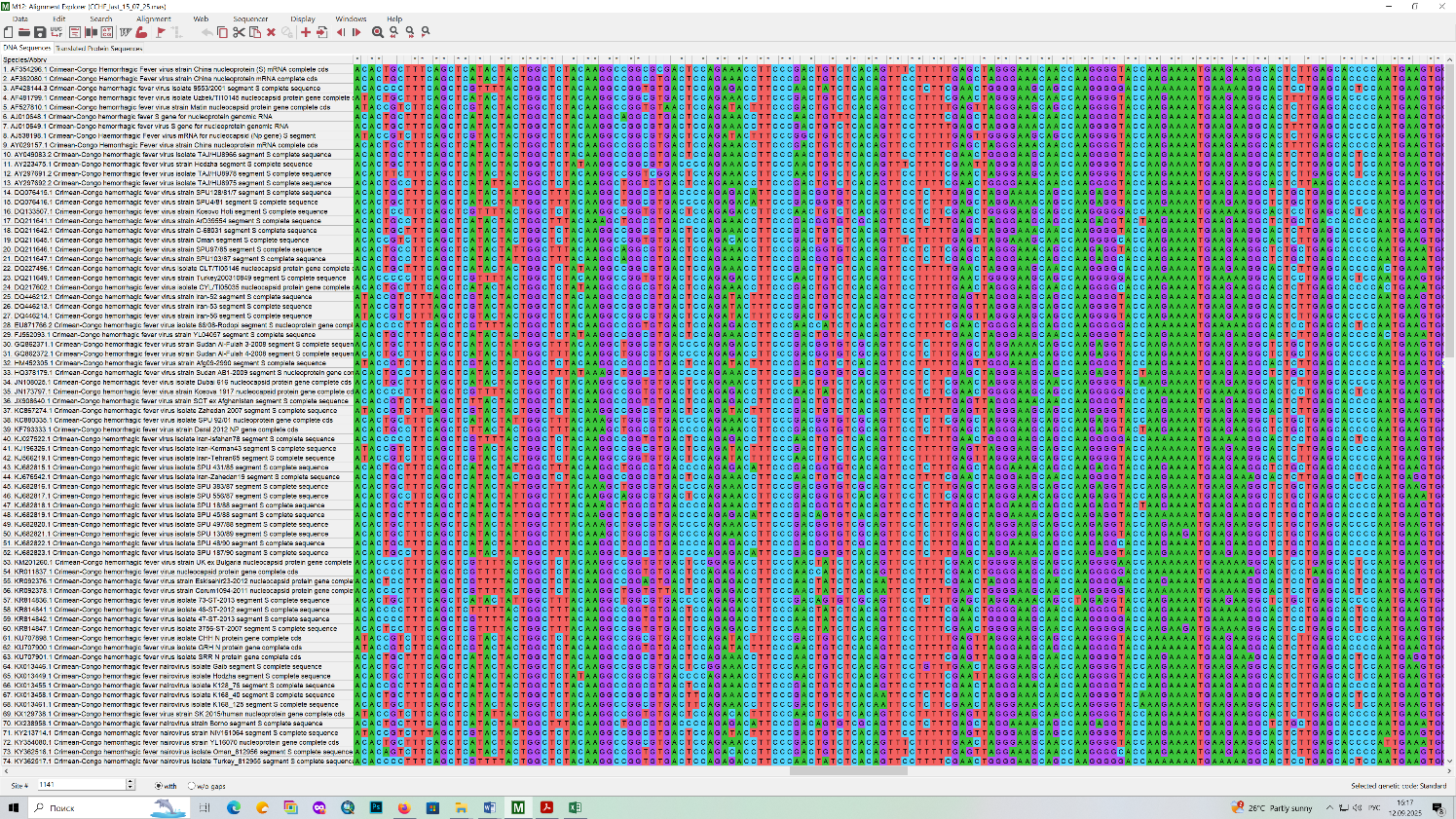


Рисунок 1 – Фрагмент множественного выравнивания нуклеотидных последовательностей S сегмента вируса *ККГЛ*


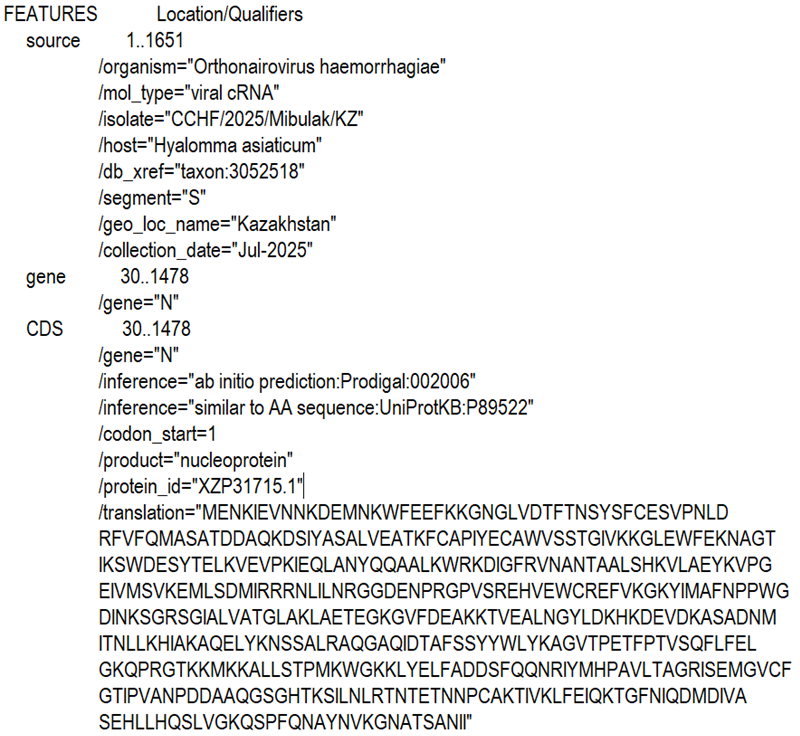


Рисунок 2 – Аннотация и аминокислотная последовательность, кодирующая NP ген S сегмента вируса ККГЛ
